# Evidence based recommendations for an optimal prenatal supplement for women in the US: vitamins and related nutrients

**DOI:** 10.1186/s40748-022-00139-9

**Published:** 2022-07-11

**Authors:** James B. Adams, Jasmine K. Kirby, Jacob C. Sorensen, Elena L. Pollard, Tapan Audhya

**Affiliations:** 1 Arizona State University, Tempe, Arizona USA; 2 University of Arizona College of Medicine, Tucson, Arizona USA; 3 Health Diagnostics and Research Institute, South Amboy, New Jersey USA

**Keywords:** Pregnancy, Vitamins, Prenatal Supplements, Folate, Vitamin B12, Vitamin D

## Abstract

The blood levels of most vitamins decrease during pregnancy if un-supplemented, including vitamins A, C, D, K, B1, B3, B5, B6, folate, biotin, and B12. Sub-optimal intake of vitamins from preconception through pregnancy increases the risk of many pregnancy complications and infant health problems. In the U.S., dietary intake of vitamins is often below recommended intakes, especially for vitamin D, choline and DHA. Many studies suggest that insufficient vitamin intake is associated with a wide range of pregnancy complications (anemia, Cesarean section, depression, gestational diabetes, hypertension, infertility, preeclampsia, and premature rupture of membranes) and infant health problems (asthma/wheeze, autism, low birth weight, congenital heart defects, intellectual development, intrauterine growth restriction, miscarriage, neural tube defects, orofacial defects, and preterm birth). The primary goal of this paper is to review the research literature and propose evidence-based recommendations for the optimal level of prenatal supplementation for each vitamin for most women in the United States. A secondary goal was to compare these new recommendations with the levels of vitamins in over 180 commercial prenatal supplements. The analysis found that prenatal supplements vary widely in content, often contained only a subset of essential vitamins, and the levels were often below our recommendations. This suggests that increasing prenatal vitamin supplementation to the levels recommended here may reduce the incidence of many pregnancy complications and infant health problems which currently occur.

**Supplementary Information:**

The online version contains supplementary material available at 10.1186/s40748-022-00139-9.

## Introduction

Vitamins are by definition essential for optimal health and development, and a deficiency of any one vitamin can lead to serious illness Although a very healthy diet rich in vegetables, fruits, whole grains, protein, and healthy fats can provide sufficient amounts of most vitamins, analysis of the National Health and Nutrition Examination Survey (NHANES) finds that diet quality in the United States is generally “poor” [1]. During pregnancy, there are increased nutritional demands including an increased need for vitamins to promote a healthy pregnancy and a healthy baby [2]. The blood levels of many vitamins decrease during pregnancy unless supplemented [2]. Therefore, prenatal supplements are necessary to assure adequate intake during preconception, pregnancy and breastfeeding.

The US Food and Drug Administration (FDA) has established Recommended Dietary Allowances (RDA) for total vitamin intake from food and supplements, but there is no national consensus on the optimal level of most vitamins for a prenatal supplement. Therefore, there is a wide variation in the content of prenatal supplements on the market today.

Pregnancy complications are common in the US, as shown in Table 1 [3–11], and many children born in the US have significant health problems, as shown in Table 2 [12–18]. This paper reviews the evidence that low levels of vitamin intake during pregnancy contributes to many of these problems, and that appropriate prenatal vitamin supplementation may reduce their risk.

**Table 1:** Rates of Pregnancy and Birth Complications in the US

Complication	Rate
Infertility (women)	6%
Miscarriages	15–20% ^a^
Gestational diabetes	8%
Preeclampsia	4%
Iron deficiency anemia in mother	27.5% in third trimester
Caesarean sections	32%
Still births	1%
Low birth weight	8%
Preterm births	10%
Postpartum depression	11.5%

^a^ The estimated rate of miscarriages after a woman knows she is pregnant is 15–20%, but the actual number of fertilized eggs that spontaneously abort is estimated to be up to 50%

**Table 2:** Incidence of some mental and physical health disorders in children in the US

Disorder	Incidence
Autism	2%
Birth Defect (heart, other)	3%
ADHD	9.4%
Learning Disabilities	8%
Asthma	7.5%
Food Allergies	5.1%
Skin Allergies	12.5%
Respiratory Allergies	17.0%
Childhood Obesity	18.5%

The purpose of this paper is to review the literature and propose evidence-based recommendations for the optimal level of prenatal supplementation for each vitamin and related nutrients (choline, inositol, and DHA) for most pregnant women in the United States. This paper will discuss the evidence that insufficient intake of vitamins during preconception and pregnancy is a contributing factor to many pregnancy/birth complications and childhood health disorders, and the evidence that optimal prenatal vitamin supplementation can significantly reduce the risk of many of those disorders. This paper proposes evidence-based recommendations for the optimal level of each vitamin, and compares those recommendations against the levels in over 180 prenatal supplements. A similar review of recommendations for prenatal mineral supplementation has been recently published [19].

It should be noted that the literature review is based on worldwide studies, but the recommendations are based in part on the NHANES data of daily intake by women in the US, so the recommendation are for women in the US. Similar recommendations could be made for other parts of the world if their average daily intake of vitamins is known.

## Methods

In this paper, we focus on 13 vitamins and three related nutrients (choline, inositol, and DHA) and each vitamin/nutrient is reviewed in a separate section. Each section includes background about that vitamin or nutrient, a summary of research, daily dietary intake (as estimated from the National Health and Nutrition Examination Survey—NHANES), Recommended Dietary Allowance, a discussion of the research, a recommendation based on our interpretation of all this data, and statistics on prenatal supplements currently on the market.

Since the research literature is vast, a systematic review of all studies would require a separate paper on each vitamin or nutrient. Instead, we provide a summary of the most relevant articles that we found from keyword searches of PubMed and forward/backward citation searches, and include a discussion of over 200 articles in this review – see Supplemental Table 1 for a summary of the articles included in this review. The primary focus of this review was on articles that provided insight into optimal dosage such as treatment studies on the effects of different doses on outcomes and biomarkers. Greater consideration was given to larger studies with a more rigorous design such as randomized, double-blind, placebo-controlled studies. When available, we included meta-analyses and systematic reviews of the literature; however, the limitation of those studies was that they generally asked whether or not a symptom was related to a vitamin deficiency or improved due to vitamin supplementation but generally did not attempt to estimate the optimal level of supplementation. The types of articles reviewed generally fell into three categories: (1) the associations of low levels of vitamins with health problems, (2) studies of changes in vitamin levels during pregnancy if un-supplemented or supplemented, and (3) clinical trials on the effect of vitamin supplementation on health problems. Each of these three areas involved separate searches for each vitamin/nutrient, using the keywords “pregnancy, name of the nutrient, and a keyword for the topic, such as blood level, clinical trial, specific health problem, meta-analysis.” Searches were included from any country, although the discussion and recommendations focus more on data from the US if available. No restriction was placed on year of the study, but more recent studies were given higher priority in the review and discussion, and most studies were from 1990 and later. In addition, some articles were found by reviewing those cited by an identified study, and also by forward literature search on key articles. Due to the vast scope of literature on the subject, a full systematic review is far beyond the scope of any single paper, but we believe that the present paper with over 200 articles cited in Supplemental Table 1 provides a broad overview of the field, and can serve as a starting point for future systematic reviews of each individual nutrient.

The NHANES data listed in this paper is for dietary intake only (not supplements) of each nutrient, since we assume that most women will stop other vitamin/mineral supplements when they start a prenatal supplement. We reported the data for women ages 20–39 years, since that is the most common time for pregnancy, and averages for other ages are generally similar. We used the 2017–2018 NHANES data for the nutrients reported then, and otherwise report the 2009–2010 data. The NHANES data on dietary intake for each vitamin/nutrient is useful for comparing to the RDA, to determine if the average intake is sufficient for most women. However, it is important to note that we report only the averages, and some women have higher or lower intake.

Note that the RDA is based on the levels required to meet the nutritional needs of 97.5% of healthy individuals, as opposed to the Estimated Average Requirements (EAR) which is set at the level needed to meet the needs of 50% of the population. Therefore, we focus on the RDA, since our interest is in meeting the nutritional needs of most pregnant women. In the cases where the RDA is higher than the NHANES intake, the difference provides an estimate of the needs for nutritional supplementation during pregnancy. However, in some cases a review of the literature suggests that higher levels are needed to reduce the risk of pregnancy complications and infant health problems.

The ultimate goal of this review is to propose evidence-based recommendations for the optimal level of each vitamin for a prenatal supplement based on currently available information, with the understanding that further research is needed for most vitamins to fine-tune our recommendations. A key point is trying to balance the benefit of additional supplementation for those women with the lowest levels of vitamins vs. the risk of adverse effects for women with the highest levels of vitamins. No single formulation is ideal for every person. However, because personalized testing to determine individualized prenatal supplementation is rare, we believe it is important to develop evidence-based recommendations for the general population while encouraging physicians and nutritionists to personalize recommendations to the extent possible.

In most cases our recommendations are for a constant amount of nutrient supplementation during pregnancy, as the effect of varying dosage during pregnancy has generally not been explored. However, for iron and choline we provide recommendations on increasing levels of supplementation during pregnancy, for the reasons discussed in those sections.

We also report on the quality of evidence and quality of the recommendations for each nutrient, using the Grading of Recommendations Assessment, Development and Evaluation (GRADE) system. GRADE evaluates the quality of evidence on a scale of very low/low/moderate/high, and evaluates the strength of recommendations as strong or weak (or none if not recommended). For example, randomized clinical trials are generally rated as high, and observational studies as low, subject to further criteria. The strength of recommendations is based primarily on four factors, including the balance of benefit vs adverse effects, the quality of the evidence, uncertainty in the relative value of different benefits and adverse effects, and cost of the treatment. In general, adverse effects of supplementation at our recommended levels is not significant, and the cost of prenatal supplements is low compared to costs of treating adverse effects such as pre-term birth. So, the strength of recommendations in this report was primarily determined by the potential benefit of supplementation and the quality of evidence. A “strong” recommendation means that we are confident the benefit of supplementation outweighs the risk, whereas a “weak” recommendation means that the benefit probably outweighs the risk.

A comprehensive list of 188 prenatal supplements currently on the market was created primarily using two databases created by The National Institutes of Health (NIH): The Dietary Supplement Label Database (DSLD) and DailyMed. Although both databases include an extensive list of prenatal supplements, some products listed are outdated and can no longer be purchased or have changed ingredients. Therefore, the list was updated using information on manufacturer websites (when available) or from labels on retail websites such as Amazon. The contents of these prenatal supplements were then analyzed and compared against the evidence-based recommendations proposed here.

Tables 3 and 4 provide a list of the pregnancy complications and infant health conditions, respectively, associated with one or more nutrients. Tables 5 and 6 show the same information, but organized by nutrient instead of by health condition.

**Table 3:** Relationship of maternal health problems to vitamin status. A “M” is added to studies which are meta-analyses or systematic reviews

Maternal Outcome	Substantial Evidence	Limited Evidence
Abdominal Pain		**Vitamin C** (Rumbold 2008) [20]
Anemia	**Riboflavin**(Ma 2008, Suprapto 2002) [21, 22]; **Vitamin A** (Thorne-Lyman 2012 M) [23]	
Cesarean Section	**Vitamin D** (Merewood 2009, Wagner 2016) [24, 25]	
Dental Decay		**Pyridoxine** (Rumbold 2008) [20]
Depression	**DHA** (Hibbeln 2002, Lin 2017 M, Zhang 2020 M) [26–28]	
Eclampsia		**Vitamin A** (Ziari 1996) [29]; **Vitamin E** (Ziari 1996) [29]
Gestational Diabetes	**Cobalamin** (Yajnik 2008, Finkelstein 2015) [30, 31] **DHA** (Goa 2020) [32]	**Inositol** (Corrado 2011) [33]
Glucose Levels	**Inositol** (Corrado 2011, Papaloe 2011) [33, 34]	
Hospitalization		**Vitamin C** (Hans 2010) [35]
Hyperglycemia		**Vitamin E** (Ley 2013) [36]
Hypertension	**Vitamin D** (Rumbold 2008, Wagner 2013) [20, 37]	**Riboflavin** (Elsen 2012) [38]
Infection		**Vitamin D** (Wagner 2013) [37]
Infertility	**Cobalamin** (Jackson 1967, Hall 1968, Bennett 2001) [39–41]	**Inositol** (Carlomagno 2011, Papaloe 2011) [34, 42]
Insulin Resistance		**Vitamin E** (Ley 2013) [36]
Megaloblastic Anemia		**Folate** (Lassi 2013 M) [43]
Nausea/Vomiting during Pregnancy		**Pyridoxine** (Chittumma 2007) [44]
Night Blindness	**Riboflavin** (Christian 1998, Graham 2007) [45, 46]	**Vitamin A** (Christian 1998) [46]
Pernicious Anemia		**Cobalamin** (Jackson 1967, Hall 1968) [39, 40]
Polycystic Ovarian Syndrome		**Inositol** (Papaleo 2011) [34]
Preeclampsia	**Cobalamin** (Mardali 2021) [47]; **Vitamin D** (Bodnar 2007, Baca 2016, Haugen 2009, & Wagner 2013) [37, 48–50]; **DHA** (Bakouei 2020 M, Kulkarni 2010, Middleton 2018) [51–53]	**Pyridoxine** (Hillman 1962) [54]; **Riboflavin** (Elsen 2012) [38]; **Vitamin A** (Ziari 1996) [29]; **Vitamin C** (Chappell 2002) [55] **Vitamin E** (Ziari 1996) [29]
Premature Rupture of Membranes (PROM)	**Vitamin C** (Rumbold 2015 M, Casanueva 2005, Ghomian 2013, Zamani 2013 & Kiondo 2014) [56–61]; **Vitamin E** (Rumbold 2015b M) [56, 60]	
Secondary Hyperparathyroidism		**Vitamin D** (Yu 2009) [62]
Urinary Tract Infection		**Vitamin C** (Ochoa-Brust 2007) [63]

**Table 4:** Relationship of infant health problem to maternal vitamin status

Infant Outcome	Significant Evidence	Limited Evidence
Alzheimer’s		**Choline** (Strupp 2016) [64]
Asthma/Wheeze	**Vitamin D** (Beckhaus 2015, Zosky 2014, Wolsk 2017) [65–67]; **Vitamin E** (Beckhaus 2015 M) [65]	**Vitamin C** (McEvoy 2014) [68]; **DHA**
Autism	**Folate** (Li 2019) [69]; **Vitamin D** (Vinkhuyzen 2018, Fernell 2015) [70, 71]	**Vitamin B12 (**Raghavan 2018, Hollowood 2020) [72, 73]
Birth Weight	**Pantothenic Acid** (Baker 1977, Haggarty 2009, Lagiou 2005, Watson 2010) [74–77]; **Pyridoxine** (Chang 1999, Ronnenberg 2002) [78, 79]; **Cobalamin** (Finkelstein 2015, Rogne 2017 M) [31, 80]	**Vitamin C** (Haggarty 2009) [74]; **Thiamine** (Bakker 2000) [81]; **Niacin** (Baker 1977) [75]; **DHA** (Carlson 2013) [82],
Cardiovascular malformation		**Pyridoxine** (Czeizel 2004) [83]
Congenital Heart Defects	**Cobalamin** (Finkelstein 2015 R, Shaw 2010) [31, 84]; **Vitamin E** (Smedts 2009, Shaw 2010) [84, 85]	**Vitamin A** (Shaw 2010) [84]; **Niacin** (Shaw 2010) [84]; **Riboflavin** (Shaw 2010) [84]
Down Syndrome		**Choline** (Strupp 2016) [64]
Gestational Length		**DHA** (Carlson 2013, Ciesielski 2019, Harris 2015, Middleton 2018 M, Miller 2006, Olsen 2000, Smuts 2003) [51, 82, 86–89, 90]
Hyperbilirubinemia		**DHA** (Goa 2020) [32]
Insulin resistance		**Cobalamin** (Finkelstein 2015) [31]
Intellectual Development	**DHA**	**Cobalamin** (Finkelstein 2015) [31]
Intrauterine Growth Restriction	**Cobalamin** (Finkelstein 2015 R) [31]	**Thiamine** (Heinze 1990) [91]
Language Difficulties		**Vitamin D** (Whitehouse 2012) [92]; **Folate** (Roth 2011) [93]
Leanness		**DHA**
Lung Function		**Vitamin A** (Checkley 2010) [94]; **Vitamin C** (McEvoy 2014) [68]
Memory		**Choline** (Boeke 2013) [95], **Cobalamin** (Finkelstein 2015) [31]
Miscarriage	**Cobalamin** (Reznikoff-Etiévant 2002 M, Hubner 2008) [96, 97]	**Vitamin D** (Andersen 2015) [98]
Neonatal Care Admissions		**DHA** (Middleton 2018) [51]
Neural Tube Defects	**Cobalamin** (Finkelstein 2015 R, Mills 1995, Molloy 2009, Ray 2007, Wald 1996) [31, 99–102]; **Folate** (Berry 1999; Czeizel 1992; Goh 2006 M, Kirke 1992; Laurence 1981; MRC 1991; Toriello 2005; Vergel 1990; Werler 1993, Wilson 2015) [103–112]; **Inositol** (Greene 2016, Greene 2017, Guan 2014, Cavalli 2011) [113–116]	**Choline** (Shaw 2010); **Niacin** (Groenen 2004); **Thiamine** (Chandler 2012) [84, 117, 118]
Neurodevelopmental Behavior Problem		**Pyridoxine** (McCullough 1990) [119]
Orofacial Defects	**Folate** (Goh 2006 M) [111]	**Vitamin A** (Krapels 2004) [120]; **Vitamin C** (Krapels 2004) [120]; **Vitamin E** (MISSING, Krapels 2004) [120]; **Biotin** (Takechi 2008) [121]; **Pyridoxine** (Krapels 2004) [120]
Learning Disabilities		**Thiamine** (Bell 1979) [122]
Perinatal Death		**DHA** (Middleton 2018) [51]
Placental Abruption	**Vitamin E** (Rumbold 2015a R) [56, 60]	
Preterm Birth	**DHA** (Carlson 2013, Ciesielski 2019, Harris 2015, Middleton 2018 M, Miller 2006, Olsen 2000) [51, 82, 86–88, 90]; **Vitamin D** (Wagner 2013, Wagner 2016) [24, 37]; **Vitamin E** (Bártfai 2012) [123]; **Cobalamin** (Rogne 2017 M) [80]	**Pyridoxine** (Ronnenberg 2002) [78]; **Folate** (Li 2019) [69]
Psychomotor Scores/Skills		**DHA** **Vitamin D** (Morales 2012) [124]
Risk for Serious Birth Defects	**Folate** (Goh 2006 M) [111]	**Riboflavin** (Robitaille 2009) [125]
Small for Gestational Age	**Cobalamin** (Finkelstein 2015 R) [31]; **Folate** (Hodgetts 2015 M) [126]	
Vitamin K Deficient Bleeding (Often intercranial hemorrhage)	**Vitamin K** (AAP 2015, Crowther 2001) [127]

**Table 5:** Relationship of vitamins to maternal health problems

Vitamin	Significant Evidence	Limited Evidence
Vitamin A	**Anemia** (Thorne-Lyman 2012 M) [23]	**Night Blindness** (Christian 1998) [46]; **Preeclampsia** (Ziari 1996) [29]; **Eclampsia** (Ziari 1996) [29]
Vitamin C	**Premature Rupture of Membrane** (Rumbold 2015 M, Casanueva 2005, Ghomian 2013, Zamani 2013, Kiondo 2014) [56–59]	**Abdominal Pain** (Rumbold 2008) [20]; **Hospitalizations** (Hans 2010) [35]; **Preeclampsia** (Chappell 2002) [55]; **Urinary Tract Infection** (Ochoa-Brust 2007) [63]
Vitamin D	**Cesarean section** (Merewood 2009, Wagner 2016) [24, 25]; **Preeclampsia** (Bodnar 2007, Baca 2016, Haugen 2009, Wagner 2013) [37, 48–50]	**Hypertension** (Wagner 2013, Rumbold 2008) [20, 37]; **Infection** (Wagner 2013) [37]; **Secondary Hyperparathyroidism** (Yu 2009) [62]
Vitamin E	**Premature Rupture of Membrane** (Rumbold 2015b M) [56]	**Eclampsia** (Ziari 1996) [29]; **Hyperglycemia** (Ley 2013) [36]; **Insulin Resistance** (Ley 2013) [36]; **Preeclampsia** (Ziari 1996) [29]
Vitamin K		
Vitamin B1 (Thiamine)		**Glucose Tolerance** (Bakker 2000) [81]
Vitamin B2 (Riboflavin)	**Anemia** (Ma 2008, Suprapto 2002) [21, 22]; **Night blindness** (Christian 1998, Graham 2007) [45, 46]	**Hypertension** (Elsen 2012) [38]; **Preeclampsia** (Elsen 2012) [38]
Vitamin B3 (Niacin)		
Vitamin B5 (Pantothenic Acid)		
Vitamin B6 (Pyridoxine)		**Dental Decay(115),** **Nausea/Vomiting** (Chittumma 2007) [44] **Preeclampsia(115)**
Vitamin B7 (Biotin)		
Vitamin B9 (Folate)	**Megaloblastic Anemia** (Lassi 2013 M) [43]	
Vitamin B12 (Cobalamin)	**Gestational Diabetes** (Finkelstein 2015, Yajnik 2008) [30, 31] **Infertility** (Jackson 1967, Hall 1968, Bennett 2001) [39–41]; **Pernicious Anemia (**Jackson 1967, Hall 1968) [39, 40]; **Preeclampsia** (Mardali 2021) [47];	
Choline		
DHA	**Depression** (Hibbeln 2002, Lin 2017 M, Zhang 2020 M) [26–28]; **Gestation Diabetes** (Gao 2020) [32]; **Preeclampsia** (Bakouei 2020 M, Kulkarni 2010, Middleton 2018) [51–53]	
Inositol	**Glucose Levels** (Corrado 2011, Papaloe 2011) [33, 34]; **Infertility** (Carlomagno 2011, Papaloe 2011) [34, 42]	**Polycystic Ovarian Syndrome** (Papaleo 2011) [34]; **Gestational Diabetes** (Corrado 2011) [33]

**Table 6:** Relationship of maternal vitamin status to infant health problems

Vitamin	Significant Evidence	Limited Evidence
Vitamin A		**Heart Defects** (Shaw 2010) [84]; **Orofacial Defects** (Krapels 2004) [120]; **Impaired Lung Function** (Checkley 2010) [94]
Vitamin C		**Wheeze** (McEvoy 2014) [68]; **Low Birth Weight** (Haggarty 2009) [74]; **Orofacial Clefts** (Krapels 2004) [120]; **Infant Pulmonary Function** (McEvoy 2014) [68]
Vitamin D	**Autism** (Vinkhuyzen 2018, Fernell 2015) [70, 71]; **Preterm Birth** (Wagner 2016, Wagner 2015 M, Wagner 2013) [24, 128]; **Asthma/Wheeze** (Beckhaus 2015, Zosky 2014, Wolsk 2017) [65–67]	**Miscarriage** (Andersen 2015) [98]; **Language Difficulties** (Whitehouse 2012) [92]; **Psychomotor Skills** (Morales 2012) [124]
Vitamin E	**Congenital Heart Defects** (Smedts 2009, Shaw 2010) [84, 85]; **Orofacial Clefts** (MISSING, Krapels 2004) [120] **Preterm Birth** (Bartfai 2012) [123]; **Placental Abruption** (Rumbold 2015a R) [56]; **Wheeze** (Beckhaus 2015 M) [65]	
Vitamin K	**Vitamin K Deficient Bleeding (Often intercranial hemorrhage)** (AAP 2015, Crowther 2001) [127]	
Vitamin B1 (Thiamine)		**Anencephaly** (Chandler 2012) [117]; **Low Birth Weight** (Bakker 2000) [81]; **Intrauterine growth retardation** (Heinze 1990) [91]; **Learning Disabilities** (Bell 1979) [122]
Vitamin B2 (Riboflavin)		**Low Birth Weight** (Haggarty 2009) [74]; **Birth defects** (Robitaille 2009) [125]; **Heart defects** (Shaw 2010) [84]
Vitamin B3 (Niacin)		**Spina Bifida** (Groenen 2004), **Heart Defect** (Shaw 2010) [84]; **Birth Weight** (Baker 1977) [75]
Vitamin B5 (Pantothenic Acid)	**Low Birth Weight** (Lagiou 2005, Watson 2010, Haggarty 2009, Baker 1977) [74–77]	
Vitamin B6 (Pyridoxine)	**Birth Weight** (Chang 1999, Ronnenberg 2002) [78, 79]	**Orofacial Defect** (Krapels 2004) [120]; **Neurodevelopmental Behavior Problem** (McCullough 1990) [119]; **Cardiovascular Malformation** (Czeizel 2004) [83]; **Preterm Birth** (Ronnenberg 2002) [78]
Vitamin B7 (Biotin)		**Orofacial Defect (**Takechi 2008) [121]
Vitamin B9 (Folate)	**Cleft Lip/Palate Defect** (Goh 2006 M) [111] **Heart Defects** (Goh 2006 M) [111] **Limb Defects** (Goh 2006 M) [111] **Neural Tube Defects** (Berry 1999; Czeizel 1992; Goh 2006 M, Kirke 1992; Laurence 1981; MRC 1991; Toriello 2005; Vergel 1990; Werler 1993, Wilson 2015) [103–112] **Autism** (Li 2019) [69]; **Small for Gestational Age** (Hodgetts 2015 M) [126]	**Preterm Birth** (Li 2019) [69] **Severe Language Delay** (Roth 2011) [93]
Vitamin B12 (Cobalamin)	**Heart defect** (Shaw 2010, Finkelstein 2015 R) [31, 84] **Intrauterine Growth Restriction** (Finkelstein 2015 R) [31] **Low birth weight** (Finkelstein 2015, Rogne 2017 M) [31, 80] **Miscarriage** (Reznikoff-Etiévant 2002 M, Hubner 2008) [96, 97] **Neural Tube Defects** (Finkelstein 2015 R, Mills 1995, Molloy 2009, Ray 2007, Wald 1996) [31, 99–102] **Preterm birth** (Rogne 2017 M) [80] **Small for Gestational Age** (Finkelstein 2015 R) [31] **Spontaneous Abortion** (Finkelstein 2015 R) [31]	**Memory** (Finkelstein 2015) [31]; **Insulin resistance** (Finkelstein 2015) [31] **Autism** (Raghavan 2018, Hollowood 2020) [72, 73]
Choline		**Alzheimer’s** (Strupp 2016) [64] **Down Syndrome** (Strupp 2016) [64] **Memory Scores** (Boeke 2013) [95] **Neural Tube defects** (Shaw 2010) [84]
DHA	**Brain Development** **Gestational Length** (Carlson 2013, Ciesielski 2019, Harris 2015, Middleton 2018 M, Miller 2006, Olsen 2000, Smuts 2003) [51, 82, 86–90] **Macrosomia** (Goa 2020) [32]; **Preterm Birth** (Carlson 2013, Ciesielski 2019, Harris 2015, Middleton 2018 M, Miller 2006, Olsen 2000) [51, 82, 86–88, 90] **Hyperbilirubinemia** (Goa 2020) [32]	**Psychomotor skills** **Gestational Size** (Carlson 2013) [82] **Leanness** **Perinatal Death** (Middleton 2018) [51] **Neonatal Care Admissions** (Middleton 2018) [51] **Wheeze**
Inositol	**Neural tube defects** (Greene 2016, Greene 2017, Guan 2014, Cavalli 2011) [113–116] **Spina Bifida** (Groenen 2004, Guan 2014) [114]	

## Results

### Vitamin A

#### Research

Vitamin A is an important fat-soluble antioxidant. It is crucial for the growth of most cells and organs, including the eyes, heart, and lungs. Low vitamin A during pregnancy is associated with night blindness and anemia in mothers (see Table 5). For infants born to mothers with lower levels of vitamin A, there is an increased risk of severe vision problems, heart defects, orofacial defects, delayed growth, and impaired lung function (see Table 6).

Retinol levels decrease steadily during pregnancy if not supplemented [129, 130]. According to two US studies [131, 132], pregnant women are more likely to be deficient in vitamin A than healthy non-pregnant women, even after supplementation. Baker et al. [131] found that 33% of un-supplemented pregnant women in the US were vitamin A deficient, vs. 17% of women who supplemented with 4000–6000 IU; none in either group were deficient in beta-carotene. Another large US study [132] investigated supplementation with 5000 IU/day of vitamin A (50% as beta carotene). They found that despite supplementation vitamin A levels were 27% lower in pregnant women during first, second, and third trimesters compared to healthy non-pregnant controls [132].In contrast, beta-carotene levels were only slightly lower during the first trimester, and increased to slightly above normal by the end of pregnancy [132]. Overall, these studies suggest that higher levels of supplementation of vitamin A, but not carotenoids, are needed during pregnancy.

According to the World Health Organization, 4.4% of pregnant women in North and South America experience night blindness during pregnancy [133]. In Nepal, 7000 mcg/week of vitamin A reduced the occurrence of night blindness during pregnancy by 67%; beta-carotene had about half as much benefit [46]. Since night blindness still occurred in some women during the study, a higher dose is likely needed.

One small study found that women who had preeclampsia and eclampsia had much lower levels of vitamin A and beta-carotene [29].

A meta-analysis of 8 studies found that vitamin A or beta-carotene supplementation significantly improved hemoglobin levels and thus modestly reduced the risk of anemia (RR = 0.81 [0.69, 0.94]) [23].

Near the end of gestation, it is important to have adequate maternal vitamin A status to maximize the vitamin A transferred to the fetus [134]. Vitamin A stores are recommended to be replenished in late gestation to prepare for breastfeeding [135]. One study has shown that high vitamin A levels were associated with more efficient lung function of offspring [94]. Another study found that the risk of orofacial clefts was significantly lower in mothers with higher dietary intakes (1677–2019 mcg/day) of beta carotene (OR 0.6) [120]. Researchers in the US found that the lowest quartile of dietary intake of vitamin A was associated with a significantly higher risk of a serious heart defect in the offspring (OR = 3.4) [84].

#### Daily intake and RDA

The NHANES [136] study found that from 2009 to 2010, the average daily dietary intake of vitamin A of US women ages 20–39 years was 596 mcg/day, which is less than the RDA of 770 mcg for pregnant women ages 19–30 years [137]. The Tolerable Upper Limit of pre-formed vitamin A is 3000 mcg, and there is no upper limit on beta-carotene or other carotenoids.

#### Discussion

Vitamin A levels decrease during pregnancy, the average intake is below the RDA, and 2500 IU (750 mcg)/day of retinol was insufficient for women in the US to increase levels to that of non-pregnant US women. Therefore, higher levels of retinol are needed. Beta-carotene or mixed carotenoids may also be helpful, but are insufficient even at normal levels to normalize levels of retinol (active form of vitamin A).

##### Quality of evidence

High.

##### Strength of recommendation to provide vitamin A during pregnancy

Strong.

#### Recommendation

For US women, we recommend that prenatal supplements contain 1200 mcg of pre-formed vitamin A (as retinol), and 1000 mcg as mixed carotenoids (mixed carotenoids are probably preferred over beta-carotene, since human food contains a mixture of about 40–50 carotenoids, including primarily α-Carotene, β-carotene, β-cryptoxanthin, lutein, zeaxanthin, and lycopene). Giving mixed carotenoids alone is insufficient to maintain normal vitamin A levels, so it is important that about 1200 mcg be provided as pre-formed vitamin A to maintain normal vitamin A levels. Re. pre-formed vitamin A, both retinol and retinyl forms are available, but we recommend retinol since retinyl needs to be transformed into retinol. This recommendation appears likely to reduce the risk of night blindness and anemia in mothers, and may reduce the risk of vision problems, heart defects, orofacial defects, and impaired lung function in their infants.

#### Caution re. medications containing excessive vitamin A

High doses of vitamin A are used in certain medications for treating acne, psoriasis, and aging, including isotretinoin (Accutane), etretinate (Tegison), or retinol. Women should wait at least 6–12 months after stopping these medications before conceiving a child as there are concerns about these forms of vitamin A storing in the body for prolonged periods, leading to a wide array of birth defects and spontaneous abortions [138].

#### Comparison with commercial prenatal supplements

Pre-formed Vitamin A (retinol) is included in 35% of prenatal supplements ranging from 500 to 8000 IU, and the median level is 2487. IU (Q1: 1962.5/Q3: 4000). Only 13% of prenatals meet or exceed our recommendation for pre-formed Vitamin A.

Beta Carotene is included in 73% of prenatal supplements ranging from 80 to 10,000 IU, and the median level is 3040.0 IU (Q1: 2000/Q3: 4000). 34% of prenatals meet or exceed our recommendation.

### Vitamin C

#### Research

Vitamin C is an important water-soluble antioxidant, and is a co-factor for many enzymatic reactions, including the production of collagen, carnitine, and neuropeptides. During pregnancy, vitamin C is important for the growth and repair of collagen and helps maintain strong bones and teeth. A deficiency in vitamin C during pregnancy may lead to premature rupture of membranes (PROM) and preterm birth due to PROM, preeclampsia, and urinary tract infections in the mother (see Table 5). Low gestational vitamin C may cause low birth weight, orofacial clefts, and decreased pulmonary functioning for infants (see Table 6).

According to the NHANES study, vitamin C deficiency or depletion existed in 32% of women ages 25–44 in the US [139]. Vitamin C levels decrease about 30% during pregnancy if not supplemented [61]. One study measured vitamin C levels during pregnancy after supplementation with 120 mg, and found that about 10% were still deficient, suggesting more is needed [132]. Researchers [61] found that 100 mg supplementation of vitamin C was enough to maintain a constant leukocyte concentration (storage) of vitamin C, but not enough to maintain plasma concentrations. A detailed pharmacokinetic analysis by Levine et al. in 2001 of non-pregnant women found that steady-state doses of 100, 200, 400, and 1000 mg/day achieved plasma levels approximately 79%, 88%, 95%, and 97%, respectively of the dosage at 2500 mg. Similar but slightly higher percentages were found for cells (neutrophils). They recommend an RDA of 90 mg to achieve 80% of the saturated value of vitamin C in most women (not accounting for pregnancy when nutrient demands are higher).

A Cochrane meta-analysis [56] found that vitamin C supplementation alone was associated with a 34% reduced risk of preterm PROM (RR 0.66, 1282 participants from five studies) and 45% reduced risk of term PROM (RR 0.55, 170 participants). Preterm PROM is important because about 1/3 of all preterm births are due to this pregnancy complication. This review found that vitamin C only reduced the risk of PROM, but not the risk of preterm birth or other pregnancy outcomes. Two of the studies which found an effect on PROM involved doses of 100 mg/day [57, 61], and two studies that used higher doses (500–1000 mg) found non-significant lower rates of PROM [58, 59]. So, 100 mg/day seems sufficient to reduce the risk of PROM, and much higher doses are probably not better.

A study in Uganda [35] found that 400 mg of vitamin C significantly reduced hospitalization during pregnancy (42% vs. 28% for placebo), where hospitalization during pregnancy is common (primarily for anemia and respiratory infections). In Mexico, researchers [63] found that 100 mg/day of vitamin C significantly reduced the rate of urinary tract infections during pregnancy (13% vs. 29%, *p* = 0.03).

Another meta-analysis [20] of 10 trials of antioxidants (mostly combined vitamin C and E) found no significant difference between treatment and control groups for the risk of preeclampsia, severe preeclampsia, preterm birth, small-for-gestational-age infants, or any baby death. The treatment group were more likely to report abdominal pain late in pregnancy (RR 1.61; one trial, 1745 women), need antihypertensive therapy (RR 1.77; two trials, 4272 women), and need hospital admission due to hypertension (RR 1.54, 95% CI 1.00 to 2.39; one trial, 1877 women). So, vitamin C therapy alone seems more helpful than vitamin C combined with alpha-tocopherol; we hypothesize that the problem may be due to the use of only alpha-tocopherol, instead of a mixture of tocopherols. However, another study [55] of 160 women at high risk for preeclampsia found a much lower risk of preeclampsia in the group supplemented with vitamins C and E, compared to the placebo group (8% vs. 26%, respectively). Another double-blind multicenter trial (17 centers in Canada and 10 in Mexico) of 2647 women found that daily treatment of Vitamin C (1 g) and Vitamin E (400 IU) did not affect gestational hypertension or preeclampsia, but increased the risk of fetal loss or perinatal death as well as preterm prelabor rupture of membranes [140]. So, these studies provide additional evidence that the combination of high-dose vitamin C and high-dose vitamin E are not helpful and are likely harmful.

In regard to infant outcomes, researchers [68] found that 500 mg/day of vitamin C improved infant pulmonary function and significantly decreased wheezing through age 1 year. The risk of orofacial clefts was significantly lower in mothers with dietary intakes of 110–129 mg/day of vitamin C (OR 0.4) or 129–300 mg/day (OR 0.6) [120]. Children with birth weight in the lowest decile were associated with women consuming diets low in vitamin C (OR 0.79, *P* = 0.028) [74].

#### Daily intake and RDA

The NHANES[49) study found that from 2017 to 2018, the average daily dietary intake of vitamin C of US women aged 20–39 was 71 mg/day. The current RDA is 85 mg/day for pregnant women[50). The Tolerable Upper Limit for pregnant women is 2000 mg/day.

#### Discussion

Vitamin C levels decrease significantly during pregnancy unless supplemented, and average dietary intake is slightly below the RDA. 32% of women in the US have vitamin C deficiency or depletion. Two supplementation studies found that 100–120 mg/day was not quite sufficient during pregnancy to normalize biomarkers of insufficiency. Dosages of 100–1000 mg/day were effective for treating PROM, a dosage of 100 mg/day reduced risk of urinary track infections, a dosage of 400 mg/day reduced risk of hospitalization, and 500 mg/day improved pulmonary function. Altogether, the data suggests that 100 mg/day is effective, and somewhat more may be beneficial.

##### Quality of evidence

High.

##### Strength of recommendation to provide vitamin C during pregnancy

Strong.

#### Recommendation

For US women, we recommend that prenatal supplements contain approximately 200 mg of vitamin C. This recommendation appears likely to reduce the risk of premature rupture of membranes and may reduce the risk of anemia, preeclampsia, urinary tract infections, and orofacial clefts, and may improve pulmonary function in infants.

#### Comparison with commercial prenatal supplements

Vitamin C is included in 96% of prenatal supplements; when included, the median level is 100 mg (Q1: 60/Q3: 120). Only 8% meet or exceed our recommendation for Vitamin C.

### Vitamin D

#### Research

Vitamin D is important for bone growth and immune function, together with vitamin K2. Low vitamin D can cause growth delays and bone deformation (rickets). Vitamin D deficiency during pregnancy is associated with a higher risk for miscarriage, preterm birth, and C-section, and a higher risk of the child developing asthma, language difficulties, and autism (see Tables 5 and 6). Supplementing with additional vitamin D during pregnancy reduces the incidence of preeclampsia, preterm birth, infection, hypertensive disorders in pregnancy, and secondary hyperparathyroidism, and increases infant mental and psychomotor scores.

Vitamin D levels decrease substantially at the start of pregnancy if not supplemented, and remain low during pregnancy [129]. One study of 494 pregnant women in the southern part of the US at less than 14 weeks gestation measured 25 hydroxyvitamin D levels by radioimmunoassay and found that 41% of pregnant women were deficient (25(OH)D levels < 20 ng/mL) in addition another 41% were insufficient (25(OH)D levels 20–32 ng/mL) [141]. The rate of vitamin D deficiency/insufficiency was highest in African Americans (97%) and Hispanic women (81%) and lowest in Caucasian women (67%). Low vitamin D during pregnancy is strongly associated with birth complications and gestational disorders for the mother if not corrected. A vitamin D deficiency is linked to: a greater than double the risk of a miscarriage in the first trimester [98]; tripling the risk of preterm birth if low in the 3rd] trimester (*p* = 0.01) [24]; double the risk of preeclampsia [48, 49]; and increased risk of C-Sect. [25]. Vitamin D supplementation of 400–600 IU/day during pregnancy has been shown to significantly reduce the risk of preeclampsia by 29% after cofounder adjustment [50]. Supplementation with 800 IU/day greatly decreased the rate of maternal secondary hyperparathyroidism, from 27% of women to 10% [62]. Supplementation of 2000–4000 IU/day resulted in higher blood levels than just 400 IU/day, and higher levels of vitamin D were associated with substantially lower risks of preeclampsia, preterm birth, infection, hypertensive disorders of pregnancy, and other health problems [37]. A blood level of 40 ng/ml or higher results in a 57% lower risk of preterm birth compared to women with levels below 20 ng/ml [24].

Low vitamin D in pregnant women doubled the risk of the child developing significant language difficulties [92]. Gestational vitamin D deficiency was associated with an almost 4 times greater likelihood of autism-related traits in a large population-based sample of over 8,000 mothers [71], and a deficiency at birth was associated with an increased risk of autism in another smaller study analyzing blood samples from children with autism and their typical sibling pairs [70]. When mothers have a circulating concentration greater than 30 ng/ml of 25(OH)D, their infants have higher mental and psychomotor scores than compared to mothers with concentrations of 20 ng/ml [124].

A meta-analysis of 32 studies found that higher maternal vitamin D intake (OR = 0.58) was associated with lower odds of wheeze during childhood [65]. Another study found a causal relationship between vitamin D deficiency during pregnancy and asthma at 6 years of age, but only in boys [66]. A combined analysis of two treatment studies (using doses of 2400 IU/day and 4000 IU/day) found that maternal vitamin D supplementation significantly reduced the risk of asthma/recurrent wheeze at 0-3yrs: adjusted odds ratio (OR) = 0.74 (95% CI, 0.57–0.96), *p* = 0.02. The effect was strongest for women with initial vitamin D levels above 30 ng/ml compared to those with initial levels below 30 ng/ml, suggesting a need for levels above 30 ng/ml [67].

#### Daily intake and RDA

The NHANES [142] study found that the average daily dietary intake of vitamin D of US women aged 20–39 was 136 IU/day, which is much less than the RDA recommendation of 600 IU/day for pregnant women [143]. Women receive about 26% of their vitamin D from their diets. The Tolerable Upper Limit is 4000 IU/day [143]. Note that vitamin D is also produced by the body after exposure to direct sunlight, but clothing, sunscreen lotion and, windows block the part of the sunlight needed to produce vitamin D. Thus, many people receive insufficient vitamin D from sunlight, especially those that are darker-skinned, have less exposure to direct sunlight or live farther from the equator, so they are at greater risk of vitamin D deficiency.

#### Discussion

Vitamin D levels decrease significantly during pregnancy unless supplemented, and most US women consume much less than the RDA. Most women in the US have vitamin D deficiency/insufficiency during pregnancy, especially those with dark skin (Hispanic and Black). Supplementation of 2000–4000 IU/day resulted in higher blood levels than just 400 IU/day, and higher levels of vitamin D were associated with substantially lower risks of preeclampsia, preterm birth, infection, hypertensive disorders of pregnancy, and other health problems. The RDA is only 600 IU/day, but that seems insufficient during pregnancy.

##### Quality of evidence

High.

##### Strength of recommendation to provide vitamin D during pregnancy

Strong.

#### Recommendation

Therefore, we We recommend at least 2000–4000 IU/day, measuring blood levels of vitamin D (as 25(OH)D) and aiming for a level of at least 30 ng/ml, and preferably 40 ng/ml. Women with darker skin (Hispanic and especially Black) are at highest risk and likely to need more vitamin D.

#### Comparison with commercial prenatal supplements

Vitamin D is included in 98% of prenatal supplements; when included, the median level is 550 IU (Q1: 400/Q3: 1000). Only 6% meet or exceed our recommendation for Vitamin D.

### Vitamin E

#### Research

Vitamin E is an important fat-soluble antioxidant. In pregnancy, low vitamin E intake is associated with hyperglycemia, preterm births, preterm placental rupture of membranes (PROM), and placental abruption (see Table 5). The offspring of women who had low vitamin E levels had an increased risk of wheeze, orofacial clefts, and serious heart defects (see Table 6). There were troubling reports from several studies when very high dose vitamin E (400 IU) and vitamin C were combined, including an increase in fetal loss and perinatal death, abdominal pain, term PROM, and preterm PROM.

A study in the Netherlands found that levels of alpha-tocopherol approximately doubled during pregnancy [130]. A study in the US [132] found that supplementing with 30 IU of vitamin E was sufficient to increase levels 50% by the third trimester, which is likely beneficial since most women in the US consume only about half of the RDA. Lower vitamin E intake during the second trimester was related to hyperglycemia and insulin resistance later in pregnancy [36]. One small study found that women who had preeclampsia and eclampsia had lower vitamin E levels [29].

A large non-randomized population-based study found that pregnant women consuming high doses (about 450 mg/day, or about 675 IU/day) of vitamin E had a lower rate of preterm births (6.6% vs. 9.3%) than those not consuming high-dose vitamin E [123]. A similar analysis found that if a woman had preeclampsia during pregnancy and then supplemented with very high doses of vitamin E (approximately 200–600 mg/day), there was a decreased risk in preterm births (8.6% vs. 10.4% for unsupplemented women with preeclampsia) [123]. Due to the non-randomized nature of these studies, the results need to be interpreted cautiously.

Vitamin E intake during pregnancy affects some childhood health conditions as well. A meta-analysis of 32 studies of maternal dietary intake found that higher maternal intake of vitamin E (OR = 0.6, 95% CI = 0.46–0.78) was associated with lower odds of wheeze during childhood (but not necessarily asthma) [65]. Mothers of children with orofacial clefts had significantly lower levels of intake of vitamin E (9% lower,* P* = 0.04). Mothers with the highest dietary intake of vitamin E (15–22 mg) were 40% less likely to have a child with orofacial clefts (OR 0.6; 95% CI, 0.3–1.3, *p* = 0.14) [120].

Two studies found conflicting evidence for the role of vitamin E intake and risk of congenital heart defects (CHD). A case–control study [85] of 276 mothers of infants with congenital heart defects and 324 controls found that for the subset of mothers who did not take a prenatal with vitamin E, there was no significant effect of vitamin E intake on the risk of CHD. However, for the small subset of mothers (36 cases, 39 controls), who took a prenatal with vitamin E (of unknown amount) there was a 5–9 times higher risk of CHD if dietary intake of vitamin E was in the upper half (12.6–33.8 mg/day). Conversely, Shaw et al. [84] examined nutrient intakes of 318 mothers of infants with congenital heart defects and 700 control mothers. For the subset who did not use prenatal vitamins supplements (52 cases of dGTA, 66 cases of tetralogy of fallot (TOF), 251 controls), they found that the lowest quartile of dietary intake of vitamin E (< 11.6 mg) was associated with significantly increased risk of a d-transposition of great arteries (dGTA heart defect) (OR 3.3; 95% CI, 1.3–8.1), but no increased risk for a TOF heart defect. For the subset who did use prenatal vitamins, they did not find an increased risk of either heart defect in the highest quartile of vitamin E consumption. Due to the conflicting results of a serious heart defect, it is unclear whether supplementing with vitamin E would be beneficial or harmful.

A Cochrane meta-analysis on vitamin E reviewed 17 studies [60] using high dose alpha-tocopherol (200–800 IU), but it was given with other supplements, so it needs to be interpreted cautiously. There was a decreased risk of having a placental abruption (RR 0.64, 7 trials, 14,922 participants, high-quality evidence). There was no significant effect on the risk of stillbirth, neonatal death, preeclampsia, preterm birth, intrauterine growth restriction, or preterm PROM. However, supplementation with high dose vitamin E (400 IU) and high-dose vitamin C (1000 mg) was associated with an increased risk of term PROM (RR 1.77, 2504 participants, two trials [140, 144]. A meta-analysis of studies of supplementing with only vitamin C [56] found that it reduced the risk of preterm PROM (5 studies) and term PROM (1 study).

Another study [145] found that supplementation with high-dose vitamin E (400 IU) and vitamin C (1000 mg) increased abdominal pain (RR 1.63; 1877 participants).

One large multi-center study (2640 women) [140] investigated the effect of 1000 mg of vitamin C and 400 IU of vitamin E, and found that it did not result in any benefit compared to placebo. However, it did result in an increased risk of PROM (10.17% in the vitamin group vs. 6.15% in the placebo group; RR, 1.65; 95% CI, 1.23–2.22) and PPROM (5.97% in the vitamin group vs. 3.03 in the placebo group; RR, 1.97; 95% CI, 1.31–2.98) and an increased risk of “fetal loss or perinatal death” (1.69% vs. 0.78%; RR, 2.20), which included spontaneous abortion, stillbirth and neonatal death before discharge. This study planned to enroll 10,000 women but stopped prematurely due to the adverse outcomes.

Overall, the studies of high-dose vitamin E and vitamin C [78, 140, 144, 145] suggest that these doses are too high. Therefore, it appears that supplementation with high-dose vitamin C alone decreases the risk of term PROM, but the addition of high dose alpha-tocopherol increases the risk of term PROM.

#### Daily intake and RDA

The NHANES [136] study found that from 2009 to 2010, the average daily dietary intake of vitamin E of US women aged 20–39 was 7 mg/day, which is half of the RDA of 15 mg for pregnant women [143]. The Tolerable Upper Limit is 1000 mg [143].

#### Discussion

US women consume only about half the RDA of vitamin E, and low maternal intake is associated with increased risk of infant wheeze, orofacial clefts, and heart defects Supplementation with 30 IU of vitamin E was found to be sufficient to increase levels 50% in pregnant women in a small study. However, supplementation with high levels of vitamin E (400 IU) is linked to adverse effects and is not recommended.

##### Quality of evidence

High.

##### Strength of recommendation to provide vitamin E during pregnancy

Weak (for low dose) and not recommended (for high dose).

#### Recommendation

For US women, we recommend that prenatal supplements contain at least 19 mg of vitamin E (28.5 IU). We hypothesize that mixed tocopherols may be preferred vs. only alpha-tocopherol, since the human diet includes primarily gamma tocopherols, and gamma tocopherols have higher anti-oxidant capacity than alpha-tocopherol. So, we hypothesize that a mixture of approximately 15 mg of alpha-tocopherol and 10 mg of other tocopherols (primarily gamma) may be best. “dl” forms (synthetic forms) should be avoided since they have little biological activity, and instead only “d” forms which are from natural sources should be used. This recommendation appears likely to reduce the current rate of wheeze in children in the US, and possibly help with hyperglycemia, preterm births, and placental abruption. It is possible that higher doses may be beneficial, but more research is needed, and there appears to be harm with very high doses such as increased rate of abdominal pain or PROM, fetal loss and perinatal death, and congenital heart defects, although the research is inconsistent. More research is needed on the effect of low dose vitamin E supplementation, as the Baker [132] study found that only 30 IU was sufficient to substantially increase levels of vitamin E, and all the other studies used very high doses (200–800 IU). Supplementation at the low doses recommended here may help reduce the risk of hyperglycemia, preterm births, preterm placental rupture of membranes (PROM), and placental abruption, and also decrease the risk of wheeze, orofacial clefts, and serious heart defects in their infants.

#### Comparison with commercial prenatal supplements

Vitamin E is included in 94% of prenatal supplements; when included, the median level is 30 IU (Q1: 23.6/Q3: 31.6). 61% of prenatal supplements meet or exceed our recommendation for Vitamin E. 20 had levels above 100 IU which may be a concern.

### Vitamin K

#### Research

Vitamin K aids in blood clotting and building strong bones. Vitamin K deficiency in pregnancy is common, in both the mother and infant shortly after birth. Preterm infants are especially at risk for excessive bleeding after birth, which often can result in intracranial bleeding (see Table 6). Supplementing with vitamin K right after birth is a common practice recommended by the American Academy of Pediatrics.

Infants are generally born with low vitamin K stores, and the vitamin K content of human milk is low, so vitamin K deficiency in infants is common. This can lead to a risk of intracranial hemorrhage (bleeding in the brain), which can cause serious damage and death. One study in the US found that 48% of cord blood samples tested at birth were positive for a marker of vitamin K deficiency [146], prior to injection with vitamin K. The American Academy of Pediatrics recommends 0.5–1 mg of vitamin K be injected intramuscularly at birth to all infants to prevent Vitamin K Deficient Bleeding (VKDB), (often intracranial bleeding), and recommends research on the optimal oral dosing after birth to prevent late VKDB (at 2–12 weeks of life) [147]. An injection is preferred over oral dosing due to better absorption, especially in infants with biliary atresia (low production of bile acids needed to absorb vitamin K) or similar conditions (Witt 2016). The initial vitamin K injection appears to be enough to last for about 1 month, but is insufficient and results in low vitamin K in breastfed infants by 1 month, and vitamin K levels drop even lower in following months [146]. Between 1 and 3 months, a treatment study found that infants need slightly more than 25 mcg/day to maintain normal levels [148]. Some countries like the Netherlands have used oral dosing up to 150 mcg/day [149]. One study [150] found that supplementing lactating mothers with 5 mg/day of phylloquinone was sufficient to achieve 50% of the plasma vitamin K levels of formula-fed infants (levels which are 10 × that of adults), after the infants received 1 mg of phylloquinone intramuscularly at birth – it is unclear if that much supplementation is needed.

The reason for low vitamin K levels in an infant is due to low levels of vitamin K in the mother, and very low transfer of vitamin K from the mother to the infant. A small study [151] found that 70% of Belgian women develop low vitamin K in their first trimester (average of 0.64 nmol/L, vs. a reference range for non-pregnant adults of 0.8–5.3 nmol/L). One study found that vitamin K dosing only slowly and slightly crossed the placenta, so that one or more doses of 10 mg led to only a 2 times higher level in the infant despite a 100 times higher level in the mother compared to un-supplemented controls [152]. Among pregnant women with previous bariatric surgery, 88% had low levels (since gut bacteria produce about half of a person’s normal vitamin K intake) [151]. In the bariatric surgery group, levels were measured later in pregnancy, and they remained low if they did not supplement with extra vitamin K, but those who supplemented with vitamin K (10 mg per week) had a normal or above-normal level of vitamin K.

For women at imminent risk of very preterm birth, vitamin K may reduce VKDB. A meta-analysis of 7 studies [127] found that vitamin K therapy (a dose of 5–10 mg, usually repeated) led to a significant reduction in severe brain bleeding (RR 0.58; 95% CI 0.37 to 0.91) and a non-significant reduction of brain bleeding (risk ratio (RR) 0.76; 95% confidence interval (CI) 0.54 to 1.06). There was speculation that the decrease of vitamin K levels during pregnancy is protective to regulate growth [153] and prevent the growth of cancerous cells [154]. However, a meta-analysis of 6 studies of vitamin K supplementation studies found no risk of childhood cancer associated with infantile supplementation with vitamin K [155].

In general, we speculate that instead of waiting until birth, it may be beneficial to provide some vitamin K supplementation during pregnancy, in addition to injections at birth, since a study of lactating mothers found that 5 mg/day resulted in a substantial increase in vitamin K levels in their breastmilk. However, research is needed to determine if this level of prenatal supplementation is beneficial or not.

#### Intake

The NHANES [142] study found that from 2017 to 2018 the average daily intake of vitamin K of US women aged 20–39 was 146 mcg/day, which is somewhat more than the RDA recommendation of 90 mcg/day for pregnant women [142]. Vitamin K is well-tolerated even at high doses, and no Tolerable Upper Limit has been established.

#### Discussion

Vitamin K intake is somewhat above the RDA, but levels decrease substantially during pregnancy (limited evidence), so modest supplementation may be useful to keep levels constant during pregnancy. However, clinical trials are needed to determine if modest supplementation is beneficial. Since transport of vitamin K from mother to fetus is minimal, vitamin K injections to the baby upon birth are needed, and there is extensive evidence of their benefit.

##### Quality of evidence

Low (for vitamin K during pregnancy).

##### Strength of recommendation to provide vitamin K during pregnancy

Weak.

#### Recommendation

We recommend that prenatal supplements contain at least 90 mcg of vitamin K, but research is needed to determine if higher levels are needed, since most infants are born with insufficient levels of vitamin K.

Also, in women of imminent risk of preterm birth, we recommend high dose maternal vitamin K therapy (10 mg, possibly repeated) to reduce the risk of severe intracranial bleeding, which can cause brain damage, including cerebral palsy, based on the meta-analysis of 8 studies.

Women with previous bariatric surgery may need 10 mg/week.

We recommend following the American Academy of Pediatrics recommendation of injection of 0.5–1 mg at birth, and further suggest additional supplementation of at least 25 mcg/day to the infant if the infant is breastfeeding unless the mother is highly supplemented (5 mg/day).

#### Comparison with commercial prenatal supplements

Vitamin K is included in 31% of prenatal supplements; when included, the median level is 90 mcg (Q1: 52.5/Q3: 90) of 5 ± 200 mcg. Only 16% meet or exceed our recommendation for Vitamin K.

### B1 (Thiamine)

#### Research

Thiamine, also known as vitamin B1, helps the body metabolize food for energy and plays an important role in maintaining a healthy cardiovascular and nervous system. It is important during pregnancy to reduce the risk of maternal gestational diabetes and low-birth weight and anencephaly in infants (see Table 6).

Thiamine is a co-factor for three critical enzymes for glucose metabolism, and thiamine deficiency results in an impairment of production and secretion of insulin, resulting in a reduction of glucose utilization [81]. During pregnancy, a study in the US found an approximately 40% decrease in thiamine levels [131], and a study in the Netherlands found about a 10% decrease [130]. One study of 174 pregnant women in the US found that at birth, 53% of women not taking a vitamin supplement had a deficient level of thiamine, and 30% of women taking a supplement of 1.5–15 mg of thiamine were deficient, suggesting that significantly more than 1.5 mg of thiamine is needed [131]. One study [132] of 563 pregnant women taking a multi-vitamin containing 3 mg of thiamine found thiamine levels were approximately 31% lower than non-pregnant women not taking a vitamin supplement, and 17–20% had a thiamine deficiency; this suggests that much higher levels, perhaps 6 mg or more, are needed during pregnancy, consistent with the [131] study. Compared with other vitamins, thiamine deficiency was the second most common vitamin deficiency, after niacin deficiency, in women taking a prenatal vitamin supplement [132].

Thiamine supplementation in pregnant women improves their glucose tolerance, and stimulates intra-uterine growth, thereby preventing low birth weight [81]. Thiamine deficiency may also be a cause of intrauterine growth retardation [91]. During normal pregnancies, the thiamine values in blood cells fall in the 28th to the 39th week of gestation from 230 nmol/l to 170 nmol/l. Women with severe intrauterine growth retardation had much lower levels, 140 nmol/l in the 30th week of gestation and 130 nmol/l in the 39th week of gestation, (*p* = 0.0001 and *p* = 0.0005, respectively) [91] again suggesting that thiamine supplementation is needed during pregnancy.

Among non-users of prenatal supplements, thiamine intake in the highest quartile (above 1.67 mg) was associated with a significantly reduced risk of anencephaly (OR 0.47) [117]. Animal studies suggest that thiamine deficiency in infancy can result in permanent learning disability, even if corrected later in infancy [122].

#### Intake

The NHANES [142] study found that from 2017 to 2018, the average daily dietary intake of thiamine of US women aged 20–39 was 1.4 mg/day, which on average meets the RDA recommendation of 1.4 mg/day for pregnant women [143]. Thiamine is very safe at these dosages, and it is so safe that no tolerable upper limit has been determined.

#### Discussion

Thiamin levels decrease substantially during pregnancy unless supplemented, and half of US women develop thiamin deficiency after birth unless supplemented. One study found that doses of 3 mg were insufficient to fully prevent thiamin deficiency, so somewhat higher doses are needed.

##### Quality of evidence

Low.

##### Strength of recommendation to provide thiamine during pregnancy

Weak.

#### Recommendation

We recommend that prenatal supplements contain approximately 6 mg of thiamine, and more may be needed pending further research. Women with intrauterine growth restriction may need additional thiamine, and in those cases, we recommend checking thiamine levels in blood cells, not plasma, since plasma was not sensitive. This recommendation may reduce the maternal glucose intolerance, risk of anencephaly, and intrauterine growth restriction/low birth weight, although more research is needed to verify these effects.

#### Comparison with commercial prenatal supplements

Thiamine is included in 85% of prenatal supplements; when included, the median level is 1.8 mg (Q1: 1.6/Q3: 5). Only 16% meet or exceed our recommendation for Thiamine.

### B2 (Riboflavin)

#### Research

Riboflavin is important for the production of thyroid hormones, producing immune cells and red blood cells, and improving photoreceptor functioning. In pregnant women, riboflavin supplementation alone may prevent severe preeclampsia and hypertension (see Table 5). When riboflavin is given in conjunction with certain vitamins/minerals, it appears to increase its effectiveness, and is shown to help reduce anemia and night blindness. For infants, riboflavin deficiency may be associated with low birth weight, and an increased risk for serious birth defects (loss of limb and heart defect) (see Table 6).

A study by Baker et al. [131] in the US suggests that riboflavin levels decrease slightly (about 7%) during healthy pregnancies, similar to results of a study in the Netherlands which seemed to show only a slight decrease [129], although another study in the Netherlands found a slight increase of about 7% by the end of pregnancy [130]. A study in the US found that 3.4 mg of riboflavin during pregnancy was enough to slightly increase riboflavin levels above that of healthy-non-pregnant women, so somewhat less is needed [132]. Another study [156] of riboflavin-deficient pregnant and lactating women in Gambia found that riboflavin supplementation of 5 mg/day led to improvements in riboflavin levels within 3–6 weeks, and reduction of symptoms of riboflavin deficiency, namely angular stomatitis (inflammation at the corners of the mouth) and papular atrophy (eye damage) within 6 weeks, but a worsening of symptoms in those not receiving supplementation, suggesting that pregnancy and lactation worsened riboflavin deficiency. Bates et al. [157] found that increasing total daily intake from 0.5 to 1.5 mg/day in pregnant Gambian women was enough to partially reduce a biomarker for the need for riboflavin (EGRAC), but not enough to normalize it. Another Gambian study by Bates et al. [157] found that a total intake of 2.5 mg/day of riboflavin (0.5 mg/day from food, 2 mg/day from supplements) was sufficient for lactation – note that demands during lactation are similar [157] or somewhat less than during pregnancy [156], suggesting similar amounts or slightly more is needed during pregnancy.

Other studies demonstrate the need for much higher amounts of riboflavin to achieve significant results. One such study in Gambia [157], found that giving 15 mg of riboflavin every 10 days had some benefit compared to placebo, but symptoms of riboflavin deficiency continued to worsen during pregnancy, so higher and/or more frequent dosing may be needed. Another such study in Venezuela [38] found that 15 mg/day of riboflavin supplementation led to a significant decrease in the number of cases of severe preeclampsia, and less severe hypertensive symptoms (lower blood pressure).

When riboflavin is given with some other supplements, it appears to have a synergistic effect, increasing the effectiveness of each. In two studies where riboflavin was given in conjunction with iron-folate supplements, it increased their effectiveness in reducing anemia in pregnancy [21], 1 mg riboflavin; [22], 5 mg riboflavin). Another study found that riboflavin (6 mg) and iron, when added to vitamin A had a greater benefit than vitamin A alone in reducing night blindness [45].

For infants, low riboflavin intake was associated with low birth weight in one study [74]. In two separate studies, riboflavin intake in the lowest quartile was associated with a nearly 3 × risk of partial or complete loss of an arm or leg (OR 2.94), and significantly increased risk of a serious heart defect (OR 3.7) [84].

#### Intake

The NHANES [142] study found that from 2017 to 2018, the average daily dietary intake of riboflavin of US women aged 20–39 was 1.8 mg/day, which is slightly more than the RDA recommendation of 1.4 mg/day for pregnant women [143]. Riboflavin is very safe even at high doses, and no Tolerable Upper Limit has been established.

#### Discussion

Riboflavin levels decrease slightly during pregnancy, and on average US women consume slightly more than RDA, so only modest levels of supplementation seem to be required. Data from several studies in Gambia suggests that a total intake of 1.5 mg/day is insufficient, 5 mg is more than sufficient, and 2.5 mg seems to be sufficient. Similarly, a US study [132] found that supplementing with 3.4 mg was more than enough, suggesting that in the US, supplementation of about 2 mg is probably sufficient, even if dietary consumption is well below the average intake of 1.9 mg. The Venezuela study suggests that 15 mg provided clinical benefit (unclear if lower doses would provide that benefit).

##### Quality of evidence

Moderate.

##### Strength of recommendation to provide riboflavin during pregnancy

Strong.

#### Recommendation

For US women, we recommend that prenatal supplements contain about 2 mg of riboflavin, but some women may need as much as 2.5 mg/day if they have a very poor diet. If hypertension occurs, doses of 15 mg may be helpful, but further research is needed to determine if 15 mg has more clinical benefit than lower doses. This recommendation should reduce the risk of mouth sore, eye damage, anemia, and possibly reduce the risk of severe preeclampsia, night blindness, heart defect, loss of the infant’s arm or leg, and low birth weight.

#### Comparison with commercial prenatal supplements

Riboflavin is included in 84% of prenatal supplements; when included, the median level is 2 mg (Q1: 1.7/Q3: 5) of 0.2 ± 50 mg. 52% of prenatal supplements meet or exceed our recommendation for Riboflavin.

### B3 (Niacin)

#### Research

Niacin is needed for many functions in the body, including energy production and development of the nervous system, digestive system, and skin. Low niacin is associated with an increased risk of birth defects (spina bifida, serious heart defect) (see Table 6).

In a study of 563 pregnant women in the US, supplementation of 20 mg/day of niacin resulted in blood levels of niacin that were 29%, 35%, and 38% lower during the first, second, and third trimester respectively, compared to healthy non-pregnant controls, suggesting that much higher levels are needed during pregnancy [132]. One US study found that about 5% of unsupplemented pregnant women had levels below that of all healthy non-pregnant controls [131].

In a case–control study of 287 pregnant women, researchers found that low dietary intake of niacin (estimated from food diaries) at preconception resulted in an increased risk for spina bifida (OR = 2.5 for lowest quartile) [118], with levels below 20 mg/day having an increased risk. One study in the US found that the lowest quartile of dietary intake of niacin was associated with a significantly increased risk of a serious heart defect (OR 3.8) [84]. Another US study found that low birthweight infants had cord blood levels that were non-significantly lower (15% lower) than normal birth-weight infants [131].

#### Intake

The NHANES [142] study found that from 2017 to 2018 the average daily dietary intake of Niacin of US women aged 20–39 was 22 mg/day, which is slightly more than the RDA recommendation of 18 mg/day for pregnant women [143]. The Tolerable Upper Limit for Niacin is 35 mg/day [143].

#### Discussion

Niacin levels decrease substantially during pregnancy unless supplemented, and one large US study found that 20 mg/day was insufficient to prevent that decrease, so somewhat higher doses are probably needed.

##### Quality of evidence

Low.

##### Strength of recommendation to provide niacin during pregnancy

Weak.

#### Recommendation

We recommend that prenatal supplements contain approximately 35 mg/day, although more research is needed to verify that amount. This recommendation may reduce the risk of spina bifida and heart defects.

#### Comparison with commercial prenatal supplements

Niacin is included in 88% of prenatal supplements; when included the median level is 20 mg (Q1: 18/Q3: 20) of 1.8 ± 100 mg. 7% of prenatal supplements meet or exceed our recommendation for Niacin.

### B5 (Pantothenic acid)

#### Research

Pantothenic acid is needed to produce Coenzyme A, which has many functions in the body, including energy production from fats, carbohydrates, and protein. A deficiency of pantothenic acid during pregnancy is associated with low birth weight in offspring (see Table 6). Blood levels of pantothenic acid decrease substantially during pregnancy [158–160].

One study [158] found that pregnant women and pregnant teens have 36% lower levels of pantothenic acid in the blood than non-pregnant women. Similarly, one small US study [159] found that pregnant teenagers (*n* = 17) had about 45% lower levels of total pantothenic acid in blood compared to non-pregnant controls (*n* = 4), despite 12 of 17 of them consuming 2–5 mg/day of supplemental pantothenic acid, providing a total of dietary plus supplemental intake of about 7.2 mg/day. One US study [160] found that pantothenic acid levels in whole blood were 24% lower in the third trimester compared to non-pregnant women, none of whom were supplemented, and who had dietary intakes of about 5 mg/day.

Four studies found that low birth weight was associated with low pantothenic acid intake/levels [74, 76, 77] or low blood levels of pantothenic acid [75].

One study [132] found that 10 mg/day of calcium pantothenate resulted in slightly higher blood levels of pantothenic acid compared to non-pregnant controls, so somewhat less pantothenic acid is sufficient to maintain constant blood levels; however, since US women consume only about half of the RDA of pantothenic acid, increasing their levels somewhat above baseline is likely beneficial.

One small study [158] investigated supplementation with 60 mg of calcium pantothenate (which is 92% pantothenic acid) starting at 4.5 months gestation. Note that most pantothenic acid exists bound in blood, and only about 12% is free; both were measured. Bound pantothenic acid is important for producing co-enzyme A, and free pantothenic acid is important for transporting certain amino acids like glycine and serine into cells. Prior to supplementation, bound levels were only 64% of the level in healthy non-pregnant women. At 7.5 months, despite supplementation, bound levels were slightly lower (59% of levels in controls), but at term, they had reached normal levels (104% of levels in healthy controls). For free pantothenic acid, levels were 17% lower at 4.5 months, and after treatment, they were 2 × higher at 7.5 months and at term compared to controls. So, it appears that bound levels decrease more than free levels during pregnancy, and higher levels of supplemental pantothenic acid are needed to normalize bound levels, especially early in pregnancy.

#### Intake

The NHANES [136] study found that from 2009 to 2010, the average daily intake of pantothenic acid of US women aged 20–39 was 4 mg, substantially less than the RDA recommendation of 6 mg/day for pregnant women [143]. Pantothenic acid is very well tolerated even at high doses, and no Tolerable Upper Limit has been established for pregnant women.

#### Discussion

Pantothenic acid levels decrease substantially during pregnancy unless supplemented, and on average their intake is only 2/3 of the RDA. One study [159] found that 2–5 mg total dietary consumption was far too low, and one study [160] study found that 5 mg total dietary consumption was too low, and one US study [132] found that 10 mg/day of supplementation was optimal. One old study [158] suggested that 60 mg might be needed to normalize levels of bound pantothenate, but we suspect problems with their measurements of bound pantothenate, and their measurements of free pantothenate suggest far less is needed. So, we believe that the Baker study [132] study (conducted 40 years later) had more robust methods, suggesting that supplementation of 10 mg/day seems optimal.

##### Quality of evidence

Low.

##### Strength of recommendation to provide pantothenic acid during pregnancy

Weak.

#### Recommendation

For US women we recommend that prenatal supplements contain approximately 10 mg of pantothenic acid. This recommendation appears likely to reduce the risk of low birth weight.

#### Comparison with commercial prenatal supplements

Acid is included in 65% of prenatal supplements; when included, the median level is 7 mg (Q1: 7/Q3: 15). 42% of prenatal supplements meet or exceed our recommendation Pantothenic Acid.

### B6 (Pyridoxine)

#### Research

Vitamin B6 affects over 100 enzymatic reactions in the body, including the production of important neurotransmitters and hormones. Vitamin B6 deficiency is associated with an increased risk of preterm birth, nausea/vomiting during pregnancy, cleft lip/palate in infants, and neurodevelopmental behavior problems in infants (see Table 6). B6 supplementation may help decrease the severity of nausea, reduce the risk of cardiovascular malformation, reduce the risk of preeclampsia, and improve birth weight.

Vitamin B6 levels decrease substantially during pregnancy if not supplemented [129, 130], and decrease even if supplemented at the standard RDA level [161]. Similarly, a functional test of vitamin B6 using an erythrocyte glutamate (EGOT) ratio in unsupplemented pregnant women in the Netherlands found that the percentage of women with a functional B6 deficiency increased from 7.5% to 25% at the end of pregnancy [129]. Approximately 10 mg/day is needed to maintain B6 levels at normal (pre-pregnancy) levels [132, 161], and even then, some women had levels below the reference range for healthy unsupplemented non-pregnant women, including 17% (1st trimester), 14% (2nd trimester), and 6% (3rd trimester) [132].

B6 deficiency doubles the risk of preterm birth [78], and is associated with a much greater risk of nausea/vomiting during pregnancy [44, 162, 163]. One study of Egyptian women (who tend to have low B6) found that vitamin B6 status was the most important nutrient in affecting infant neurobehavioral development and maternal-infant interactions [119]. One study of orofacial clefts (cleft lip/palette) found that the lowest quintile of B6 intake was associated with a 61% higher risk of orofacial clefts [120]. There is limited evidence that vitamin B6 supplementation during pregnancy may help decrease the severity of nausea [44, 162–165], risk of preeclampsia [166], risk of cardiovascular malformation [83] and improve birth weight [79] – however, further studies are needed to verify these potential benefits [167]. 2 mg was found to be sufficient to improve birth weight [79]. One study of supplementation with a high dosage of vitamin B6 (20 mg/day) found that it significantly reduced the rate of dental decay during pregnancy [54].

#### Intake

The NHANES [142] study found that from 2017 to 2018, the average daily intake of vitamin B6 of US women aged 20–39 was 1.8 mg/day, which is the same as the RDA recommendation of 1.9 mg/day of vitamin B6 for pregnant women [143]. The Tolerable Upper Limit is 100 mg/day [143].

#### Discussion

Vitamin B6 levels decrease substantially during pregnancy unless supplemented, and about 10 mg/day is sufficient to maintain normal levels and prevent functional B6 deficiency.

##### Quality of evidence

Moderate.

##### Strength of recommendation to provide vitamin B6 during pregnancy

Strong.

#### Recommendation

We recommend at least 10 mg/day because that is the dosage required to keep vitamin B6 levels from decreasing during pregnancy [132, 161]. A daily dose of 10 mg may reduce the risk of nausea, preeclampsia, maternal dental decay, preterm birth, low birth weight, cleft lip/palate, and cardiovascular malformation. Much higher doses (25 mg every 8 h for 3 days) were found to decrease symptoms of nausea [164].

#### Comparison with commercial prenatal supplements

Vitamin B6 is included in 97% of prenatal supplements; when included, the median level is 5 mg (Q1: 2.5/Q3: 20). 41% of prenatal supplements meet or exceed our recommendation for Vitamin B6.

### B7 (Biotin)

#### Research

Biotin is necessary for several enzymes involved in energy metabolism from fats and carbohydrates. During pregnancy, animal studies demonstrate that biotin deficiency may result in birth defects that include malformations to the face and extremities, impaired fetal development, or miscarriage.

A study by Baker et al. [131] found that biotin levels during a healthy pregnancy were 29% lower than in healthy non-pregnant controls. Several more recent studies suggest that marginal biotin deficiency occurs in about half of pregnancies [168–170].

Biotin transport across the placenta is limited, and several animal studies found that a mild biotin deficiency in the mother led to severe biotin deficiency in her offspring, which is highly teratogenic (likely to cause birth defects or terminate the pregnancy), and that this effect was consistent across multiple animal species [169, 171, 172]. An in vitro study of biotin deficient human embryonic palatal cells demonstrates growth retardation vs. controls, further supporting the role of biotin deficiency in the formation of cleft lip palate [121]. It is important to note that the timing during gestation and amount of increase in urinary excretion of 3-hydroxyisovaleric acid (3HIA) in animals, which is a marker of biotin deficiency, was similar to the 3HIA increase that occurred spontaneously during the first trimester of human pregnancy [169, 173]. This provides an indirect, yet important association that a marginal biotin deficiency in humans may yield the same teratogenic effects that it does in animals.

One study found that biotin supplementation of 300 mcg/day for two weeks was sufficient to treat the deficiency [168]. Another study found that a diet containing 57 mcg/day for 10–12 weeks was insufficient to normalize a biomarker of marginal biotin deficiency [170]. A brief review paper [173] recommended that total biotin intake during pregnancy be in the range of 60–90 mg/day. A study by Baker [132] found that 30 mcg/day of supplemental biotin was sufficient to slightly increase levels of biotin above that of healthy controls, but they did not measure 3-HIA, a biomarker for need for biotin. Note that normal gut bacteria make a significant amount of biotin (roughly comparable to that in the human diet), so people with gastrointestinal problems may need extra biotin.

#### Intake

The NHANES study did not measure biotin. Using food intake data from the NHANES II, the mean biotin intake of young women aged 18 to 24 years was estimated to be 40 mcg/day [174], which is higher than the RDA recommendation of 30 mcg/day for pregnant women [143]. Biotin is regarded safe in high doses; therefore, no Tolerable Upper Limit has been established.

#### Discussion

Biotin levels decrease substantially during pregnancy, so although US women on averaged consume somewhat more than the RDA, about half of US pregnant women have biomarkers of mild biotin deficiency. A diet containing 57 mcg/day was insufficient to normalize biomarkers, but supplementation of 300 mcg/day for 2 weeks was sufficient. So, we estimate that steady consumption of approximately 100 mcg/day would be sufficient.

##### Quality of evidence

Low.

##### Strength of recommendation to provide biotin during pregnancy

Weak.

#### Recommendation

For US women we recommend that prenatal supplements contain approximately 100 mcg of biotin, although more research is needed. Women with bariatric surgery or major gastrointestinal problems may need an extra 50–100 mcg of biotin, since normal gut bacteria produce a significant amount of biotin, comparable to dietary intake. This recommendation may reduce the risk of miscarriages and birth defects, but more research in human pregnancy is needed.

#### Comparison with commercial prenatal supplements

Biotin is included in 72% of prenatal supplements; when included, the median level is 280 mcg (Q1: 35/Q3: 300) of 17.5 ± 3000 mcg. 43% of prenatal supplements meet or exceed our recommendation for Biotin.

### B9 folate

#### Research

Folate is important for DNA synthesis and methylation, which is important for the modulation of gene expression. Folate is also important for the metabolism of several amino acids. It is essential for normal cell growth and replication. Folate supplementation during pregnancy is proven to reduce the risk of neural tube disorders and megaloblastic anemia (see Table 6). It also reduces the rate of other birth defects, preterm birth, and (if taken preconception) small-for-gestational-age (see Table 6). Low levels of folate are associated with a greater risk of having a child with autism. High levels of unmetabolized folic acid are associated with a greater risk of autism and food allergies.

Most studies have reported that folate levels in blood decrease significantly during pregnancy unless supplemented [129, 175], although one study in the Netherlands [130] found a slight decrease in serum folate but a slight increase in RBC folate.

One study in Scotland [175] investigated prenatal supplementation of 0, 124, 355, and 530 mcg of folate in addition to an iron supplement. They found that levels of folate at the end of pregnancy were 40% lower than that of healthy non-pregnant women, and that a dose of 355 mcg was sufficient to maintain median serum folate levels at postpartum that were equivalent to that of healthy non-pregnant women, whereas lower/higher dosages results in lower/higher serum folate levels. Similarly, it was found that doses of 355 and 530 mg resulted in zero cases of megaloblastic anemia, vs. 11% in the unsupplemented group, 6% in the group that received only iron, and 2% in the group that received iron and 124 mcg of folate. Serum folate levels were much lower in the mothers who developed megaloblastic anemia than in the mothers with a healthy pregnancy, similar to several previous studies.

A meta-analysis of four studies with 3839 pregnancies [43] found that folate supplementation dramatically reduced the rate of megaloblastic anaemia (OR = 0.21), which occurs with severe deficiency of folate and/or vitamin B12.

One study [132] measured folate levels in 563 pregnant women in the US during their first, 2nd, and 3rd trimester while they were receiving 1000 mcg/day of folic acid. They found that average blood levels were 25–26 ng/ml during each trimester, compared to 10 ng/ml in healthy non-pregnant, non-supplemented women. So, folate supplementation at a level of 1000 mcg increases levels well above that of healthy non-pregnant women, consistent with the Willoughby [175] study.

The World Health Organization recommends that the optimal RBC-folate concentrations for prevention of NTD’s are > 906 nmol/L (approximately 416 mcg/L), based on a study that found a strong inverse relationship between RBC folate at 15 weeks gestation and rate of NTD’s [176]. Specifically, that study found that RBC-folate levels of < 340 nmol/L had a risk of NTD’s of 66/10,000, vs. a much lower risk of 8/10,000 in women with RBC-Folate levels of > 906 nmol/L. The WHO estimated that it required about 450 mcg/day of intake of dietary folate equivalents from natural food to achieve a level of approximately 1050 nmol/L.

Folate supplementation during pregnancy has been very firmly established as being important for reducing the risk of neural tube defects in infants, with most studies involving a dose of 400 mcg/day [103–110, 112, 177].

Folate fortification of foods has been implemented in many countries and shown to substantially reduce, but not eliminate, the risk of NTDs [110] Folate fortification began in the US in 1998. In the US, analysis of data from NHANES [178] found that folate fortification of foods increased mean red blood cell folate concentration in women ages 15–44 years from 686 ± 12 nmol/L pre-fortification (1988—1994) to 1060 ± 9 nmol/L post-fortification (1999–2010). It is important to note that approximately 1/3 of all people (men and women of all ages) evaluated in this study used supplements, and that average levels were 11% lower in non-supplement users compared to the entire group. Thus, we estimate that folate levels in women ages 15–44 years not taking supplements were approximately 943 nmol/L. It is important to note that these are averages, and median values are lower, so over half of US women are below the WHO recommendations for prevention of NTD’s without supplementation. For example, the central 95% range of RBC folate levels for non-Hispanic white women ages 20–59 ranged from 514 to 2530 nmol/L, and levels were slightly lower for Mexican American women and non-Hispanic black women. Furthermore, since folate levels decrease significantly during pregnancy, this is further reason for women to take supplemental folate during pregnancy; fortification alone is not enough for well over half of women.

The Society of Obstetricians and Gynaecologists of Canada has recommended that in countries like Canada with folate fortification of food that pregnant women supplement 400 mcg of folate if at low risk of NTDs starting 2–3 months preconception and continuing throughout pregnancy and 4–6 weeks during breastfeeding. For women at moderate or high risk of NTD’s, recommendations are 1000 mcg and 4000 mcg, respectively, during preconception and the first 12 weeks of gestation, followed by 400–1000 mcg for the remainder of pregnancy and first 4–6 weeks of lactation.

As is discussed more in other sections, the risk of neural tube defects can be further reduced if supplemented with vitamin B12 or inositol, and there is limited evidence that choline and selenium may also help reduce the risk of neural tube defects.

Low folate is also significantly associated with risk of other birth defects. A meta-analysis [111] of studies using a multi-vitamin supplement containing folic acid demonstrated not only a reduction in NTDs (OR = 0.67), but other congenital anomalies as well [111]. Analysis of case–control studies found that folate reduced the risk of heart (5 studies, OR = 0.78), cleft lip or palate (10 studies, OR = 0.76), and limb defects (2 studies, OR = 0.48).

Low folate is also significantly associated with risk of preterm birth. A meta-analysis of 27 studies [179] found that folate blood levels and dietary folate intake were associated with a lower risk of preterm birth (OR = 0.72 and 0.68, respectively). Higher folate supplementation and starting supplementation preconception were both associated with a lower risk of preterm birth.

Similarly, a large meta-analysis [126] of 108,525 pregnancies analyzed the effect of folate intake on small-for-gestational-age (SGA). They found that folate use preconception significantly reduced the risk of SGA (OR 0.80 for 10th %, *p* < 0.01; OR 0.78 for 5th %, *p* < 0.01). However, post-conceptual folate supplementation had no effect.

One large study [93] found that intake of folate or folate-containing multivitamins was associated with an approximately 50% reduction in the risk of severe language delay in children at age 3 years.

Severe language delay is a core symptom of autism, and there is a large body of recent research linking a risk for autism spectrum disorders (ASD) and timing or absence of consuming folic acid during preconception and pregnancy. Many studies have now investigated the effect of folic acid and/or multivitamin use during pregnancy on the risk of ASD in offspring. A meta-analysis of 6 prospective studies [180] found that maternal supplementation with folic acid was associated with a decreased risk of ASD in the child (RR = 0.64 (95% CI: 0.46, 0.90). However, one study [73] found that either low intake (≤ 2 times/week) or high intake (> 5 times/week) resulted in a higher risk of ASD vs. moderate intake (3–5 times/week). Similarly, they found that very high levels of maternal plasma folate at birth (> 90th %) or vitamin B12 (> 90th %) resulted in an increased risk of ASD (OR-2.5), and if both folate and folate B12 high, the risk was very high (OR = 13.7). Conversely, if both B12 and folate were low, the risk of ASD was also increased (OR = 2.4). Similarly, a study of cord blood from 92 children with ASD and 475 neurotypical found that the highest quartile of unmetabolized folic acid (UMFA) was associated with a much higher risk of ASD (OR = 2.26, CI 1.08–4.75), especially in black children (OR = 9.85, CI = 2.53,38.31) [181]. There was no significant relationship of the bioactive form of folate (5-methyltetrahydrofolate, 5-MTHF)) or total folate with risk. So, altogether this suggests that moderate folic acid intake is beneficial in reducing the risk of ASD, but excessive folic acid intake results in unmetabolized folic acid that is associated with a greater risk of ASD. This suggests that natural forms of folate, such as folinic acid or 5-MTHF, may be preferred over folic acid.

Total folate and unmetabolized folic acid have also been associated with a strong risk of food allergies. A study investigated 1394 children in the US, including 507 children with food sensitization and 78 with food allergy [182]. Maternal total folate at birth was 14% lower in the children who developed food allergies. Maternal total folate concentrations in the third quartile (30.4–44.8 nmol/L) resulted in a much lower odds of developing food allergy than those in the first quartile (6.64–19.7 nmol/L), suggesting that a moderately high, but not highest, level was optimal. In contrast, levels of unmetabolized folic acid were 32% higher in cord blood of children who developed food allergies. The highest quartile of unmetabolized folic acid in cord blood had a much higher risk of food allergy (OR = 8.5, *p* < 0.001). So, this suggests that total maternal folate is protective against food allergies, and that a decreased ability to metabolize folic acid (a synthetic form) to the active form (5-MTHF) in cord blood is associated with a much higher risk of food allergy.

#### Intake

The NHANES [142] study found that from 2017 to 2018, the average daily dietary intake of folate of US women aged 20–39 was 440 mcg/day, which is somewhat less than the RDA recommendation of 600 mcg of folate for pregnant women [143]. The Tolerable Upper Limit for folate is 1000 mcg [143].

#### Discussion

Folate levels decrease significantly during pregnancy unless supplemented, and average dietary intake of folate is about 25% less than the RDA, so folate supplementation is needed, and many studies suggest that 400 mcg/day is sufficient. However, most folate in prenatal supplements is in the form of folic acid, which is an artificial form, and excess unmetabolized folic acid is associated with increased risk of food allergies and autism. So, we recommend that folate be given as folinic acid or MTHF, although more research is needed.

##### Quality of evidence

High.

##### Strength of recommendation to provide folate during pregnancy

Strong.

#### Recommendation

For US women we recommend that prenatal supplements contain approximately 400 mcg of folate, and it should be started at conception or earlier to reduce the risk of NTD’s, small-for-gestational-age, and autism. If there was a previous birth with a neural tube defect, higher doses (around 4 mg) may be considered, and blood levels of folate and vitamin B12 should be measured. Folic acid is an artificial form of folate, and people vary greatly in their ability to convert it to the bioactive forms [183], so it appears that the natural forms of folate such as folinic acid or 5-methyl-tetrahydrofolate (5-MTHF) may be preferred, including for the prevention of autism and food allergies.

#### Comparison with commercial prenatal supplements

Folate is included in 98% of prenatal supplements; when included, the median level is 800 mcg (Q1: 400/Q3: 1000). 95% of prenatal supplements meet or exceed our recommendation for folate. 30% are at levels of 1000 mcg or above, which may be linked to a higher risk of food allergies and autism if using only folic acid. 71% of supplements use only folic acid, 13% use a combination of folic acid and MTHF, and 15% include only MTHF.

### B12 (Cobalamin)

#### Research

Vitamin B12 is involved in the formulation of red blood cells, cellular metabolism, and the synthesis of both DNA and myelin. Both folic acid and vitamin B12 are needed for recycling homocysteine to methionine, which is important for the production of SAM, the primary methyl donor in the body. It is important for reducing risk of infertility, miscarriage, gestational diabetes, preeclampsia, and preterm birth for the mother (see Table 5). For the infant, vitamin B12 deficiency is associated with low birth weight, neural tube defects, serious heart defect, and childhood diabetes (see Table 6).

Vitamin B12 levels decrease during pregnancy [129, 130, 184]. One study found that two-thirds of mothers in India had low vitamin B12 levels (< 203 ng/l) [30]- note that most people in India are vegetarians [185], and vegetarians are at the greatest risk of low vitamin B12. In contrast, in the US, vitamin B12 insufficiency among pregnant women was 21%, and 7% were classified as vitamin B12 deficient [186]; also, B12 levels in pregnant women were 20% lower than in non-pregnant women, even though most were probably taking a prenatal vitamin. In Canada, it is estimated that about 5% of pregnant women are deficient in vitamin B12 during the first 28 days of pregnancy, and 10% later in pregnancy [187]; they estimated that 35% of neural tube defects are due to vitamin B12 deficiency. Four studies [99–102] found that low vitamin B12 status was strongly associated with a substantially increased risk of neural tube defects; note that folate and vitamin B12 work together in preventing neural tube defects. Severe B12 deficiency causes pernicious anaemia, which is a known cause of infertility and miscarriage [39–41, 96]. A meta-analysis of five studies found that vitamin B12 deficiency was associated with an increased risk of miscarriage (OR = 2.5) [96]. Similarly, a later study found that women with miscarriage had much lower levels of vitamin B12 (197 vs. 300 pg/mL, *p* = 0.004) [97]. Low maternal B12 levels are correlated with a higher risk of type 2 diabetes in offspring [30]. A meta-analysis [80] of eighteen studies (11,216 pregnancies) found B12 deficiency (< 201 ng/L) was associated with a slightly higher risk of low birth weight (adjusted risk ratio = 1.15) and a slightly higher risk of preterm birth (adjusted risk ratio = 1.21). One study in the US found that the lowest quartile of dietary intake of vitamin B12 was associated with significantly increased risk of a serious heart defect (OR 4.0) [84].

A major review article [31] of 122 observational studies and 1 randomized trial found that low maternal or cord blood B12 was associated with gestational diabetes (1 study), neural tube defects (9 studies), spontaneous abortions (2 studies), low birth weight/IUGR/small for gestational age (3 studies), congenital heart defects (4 studies), poorer infant memory (1 study), excessive crying (1 study), infant/child insulin resistance (3 studies). Low B12 was possibly associated with maternal anemia (2 positive studies, 1 negative study, 1 mixed study) and recurrent abortion (2 positive studies, 1 mixed).

A meta-analysis of 19 studies found that women with preeclampsia have significantly lower levels of vitamin B12 than healthy pregnant women [47].One small study [184] found that total consumption of 2 × the RDA resulted in a 30% decrease in serum B12 levels by the third trimester compared to the first, and in the third trimester, 35% of the participants had serum vitamin B(12) concentrations < 201 ng/L. The authors argued that this temporary decrease was not harmful based on other biochemical markers of B12 status. One small study of 26 pregnant women found that providing approximately 3 × the RDA of vitamin B12 from both diet (6 mcg) and supplements (2.6 mcg) was enough to stabilize vitamin B12 levels during pregnancy [188]. However, one large study in the US found that supplementation of 12 mcg/day still resulted in levels that decreased during pregnancy and were 38% lower at the end of pregnancy compared to healthy non-pregnant controls [132]. The microbiological assay by Baker [132] is probably more reliable due to limitations of other methods of accurately extracting B12 and likely explains the difference between the studies. Therefore, substantially higher levels than 12 mcg/day appear necessary to stabilize B12 levels during pregnancy.

One large study [189] in India (where B12 deficiency is common due to vegetarian diets) found that 50 mcg/day of vitamin B12 during pregnancy and early lactation led to significant increases in maternal B12 levels in blood and breastmilk, and infant levels of B12, and improved biomarkers of infant need for B12 (homocysteine, methylmalonic acid).

Abnormal maternal vitamin B12 levels may also be linked to risk of autism. As discussed in the folate section, one study [73] found that very high levels of maternal plasma folate at birth (> 90th %) or vitamin B12 (> 90th %) resulted in an increased risk of ASD (OR-2.5), and if both folate and folate B12 high, the risk was very high (OR = 13.7). Conversely, if both B12 and folate were low, the risk of ASD was also increased (OR = 2.4). Another study of maternal blood levels at 2–5 years after birth found that mothers of children with ASD had 25% lower levels of vitamin B12 compared to mothers of typical children, *p* = 0.003; so, investigation of their B12 levels during pregnancy is warranted [72].

#### Intake

The NHANES [142] study found that from 2017 to 2018, the average daily dietary intake of vitamin B12 of US women aged 20–39 was 3.67 mcg/day, which is more than the RDA recommendation of 2.6 mcg/day for pregnant women [143]. However, a small percentage of women may need approximately 400 mcg or more, due to very poor absorption (lack of intrinsic factor needed for absorption of B12). Vitamin B12 is very well tolerated even at high doses, and no Tolerable Upper Limit has been established.

#### Discussion

Vitamin B12 levels decrease substantially during pregnancy unless supplemented at levels well above the RDA. The average dietary intake is more than the RDA, but much higher intake is needed to maintain normal blood levels.

##### Quality of evidence

Moderate.

##### Strength of recommendation to provide vitamin B12 during pregnancy

Weak.

#### Recommendation

We recommend approximately 25 mcg/day (preferably as hydroxocobalamin since it is better absorbed and has better retention) pending further research. It is important that vitamin B12 be supplemented for at least a month before conception to reduce the risk of neural tube defects, since they form in early pregnancy. Vegetarians should consume approximately 50 mcg/day due to the very low B12 content of vegetarian diets (B12 is mostly found in fish, meat, poultry, eggs, milk, and milk products). A very small percentage of women of child-bearing age may have low intrinsic factor, and without that, the absorption of vitamin B12 is only about 1%, so 100 × higher oral doses are needed than the standard RDA; i.e., about 500–1000 mcg/day for this population. This recommendation appears likely to reduce the rate of infertility, miscarriages, gestational diabetes, preeclampsia, preterm birth, low birth weight, neural tube defects, serious heart defect, and possibly type 2 diabetes in offspring.

#### Comparison with commercial prenatal supplements

Vitamin B12 is included in 97% of prenatal supplements; when included, the median level is 8.5 mcg (Q1: 8/Q3: 20). Only 23% of prenatals meet or exceed our recommendation.

### Choline

#### Research

Choline aids in the production of phosphatidylcholine (the main component of cell membranes) and acetylcholine (an important neurotransmitter involved in muscle control, memory, cognition, and cardiovascular regulation). In addition, choline is the primary dietary source of methyl groups (after it is converted to betaine), which modulates the DNA of all cells. It is important for optimal fetal brain development as well as possibly reducing the risk of neural tube defects, autism, and Down syndrome in the infant (see Table 6).

Choline is needed for optimal fetal brain development, and the majority of women are consuming too little choline [190]. There is an increased demand for choline in late pregnancy [191]. Since choline influences, several physiological systems in the infant, supplementing mothers with choline may have a long-term impact on the child’s health [192]. Higher maternal choline levels, especially in the second trimester, are associated with higher visual memory scores in their children at age 7 [95]. Also, the higher the status of choline in the mother, the greater the protective effect it had against neural tube disorders (OR = 0.14) [84]. One study found that dietary intake of 920 mg/day for 12 weeks might not be enough during pregnancy [193]. A review article suggests that maternal supplementation of choline may reduce the risk of Down’s syndrome and Alzheimer’s [64]. One study [128] that involved supplementing pregnant women with 900 mg/day of choline (as phosphatidylcholine) found it was safe, and helped increase cerebral inhibition in the infant at 5 weeks (but not at 13 weeks), which may be relevant to risk of schizophrenia.

#### Intake

The NHANES [142] study found that from 2017 to 2018, the average daily intake of Choline for US women aged 20–39 was 285 mg/day, which is substantially less than the RDA recommendation of 450 mg/day of choline for pregnant women. The Tolerable Upper Limit is 3500 mg/day.

#### Discussion

Average dietary intake of choline is much less than the RDA, and demand for choline increases as pregnancy progresses. Total dietary intake of 920 mg/day may be insufficient, but supplementation with 900 mg/day was safe and possibly beneficial.

##### Quality of evidence

Low.

##### Strength of recommendation to provide choline during pregnancy

Weak.

#### Recommendation

Therefore, for US women, we recommend that prenatal supplements contain at least 350 mg of choline during the first two trimesters, and roughly 600 mg in the third trimester, especially for women who do not consume several eggs/week (eggs have the highest dietary content of choline per serving, with one large egg containing 300 mg of choline). This recommendation appears likely to improve brain development in infants, and possibly help with other conditions as well.

#### Comparison with commercial prenatal supplements

Choline is included in 40% of prenatal supplements; when included, the median level is 25 mg (Q1: 10/Q3: 55) of 0.6 ± 550 mg. Only 2% of prenatal supplements meet or exceed our recommendation for choline.

### DHA

#### Research

Docosahexaenoic acid (DHA) is an essential part of the brain, eyes, and of the membrane of every cell. It is an essential fatty acid that needs to be consumed as part of a healthy diet. The primary source of DHA is from fish, but humans also have a limited ability to convert about 9% of alpha-linolenic acid to DHA and 21% to EPA [194]. During pregnancy, DHA is especially important for reducing the risk of preterm birth and preeclampsia, and for treating gestational diabetes (see Table 5).

A systematic review [195] of 13 studies of DHA levels during pregnancy found that absolute concentrations of DHA and other omega-3 fatty acids in blood increase during pregnancy, especially from trimester 1 to 2, presumably due to increasing need by the fetus. However, there is a decrease in the relative concentration of DHA compared to other fatty acids, due to increased transfer of DHA to the infant, especially during the 3rd trimester. Similarly, individual studies investigated other indicators of DHA status (DHA deficiency index) and also reported evidence of a steadily increasing deficiency of DHA from early in pregnancy to delivery [196, 197]. One study compared 300 mg/day and 600 mg/day of DHA vs. placebo [86] in 345 women in the US. They found that gestational length increased 3.5 days (*p* = 0.06) and 4.0 days (*p* = 0.03) in the two treatment groups compared to controls. RBC DHA decreased 10% in the controls, but increased 7% in the 300 mg/day group and 21% in the 600 g/day group. The combined 300 and 600 mg/day groups had a significantly lower rate of early preterm birth compared to the placebo group (1.7% vs. 5.7%, *p* < 0.05), and gestational length increased 3.5 days (*p* = 0.06) and 4.0 days (*p* = 0.03) in the two groups compared to controls.

Regarding perinatal depression, a meta-analysis [26] of 12 studies of omega 3 fatty acid levels in blood found that, compared to healthy controls, women with perinatal depression (prenatal or postnatal) had significantly lower levels of DHA and total n-3 PUFAs and significantly increased ratio of n-6/n-3. A subgroup analysis for women with prenatal depression found that they had significantly lower levels of n-3 PUFAs, EPA, and DHA. Both prenatal and postnatal depression subgroups had significantly higher ratio of n-6/n-3. Similarly, an ecological analysis of 22 countries [27] found that rates of postpartum depression varied widely between countries, from 2 to 24%, and higher concentrations of DHA in breastmilk and higher seafood consumption were strongly associated with lower levels of postpartum depression (*R* = -0.81, *p* < 0.001 and *R* = -0.84, *p* < 0.0001). A meta-analysis [28] of eight omega-3 supplementation studies for 638 women with perinatal depression found that supplementation had moderate benefits on reducing depression (SMD = 0.65, 95% CI = : 0.10, 1.20, *P* = 0.02). Doses were moderate to high (1–6 g/day of omega 3). The studies with a ratio of EPA/DHA above 1.5 had a higher benefit, consistent with similar studies for a major depressive disorder, which found that EPA-rich formulas with EPA above 1 g/day were most beneficial [198]. However, a meta-analysis of several prophylactic omega-3 supplementation studies did not demonstrate a significant benefit of omega 3 supplementation in preventing perinatal depression [51]; it is possible it is only beneficial in those with lower levels of omega 3 fatty acids.

Regarding preterm birth and gestational duration, one epidemiological analysis [87] investigated preterm birth (< 37 weeks gestation) and total omega 3 intake in 184 countries. The fit to the data found that rates of preterm birth were approximately 12% at the lowest level of omega 3 consumption, and decreased linearly to about 9% at 600 mg/day, and then plateaued at levels above 600 mg/day. Similarly, three small studies [199–201] found that higher levels of omega-3 fatty acids in erythrocytes (measured in mid or late pregnancy) were associated with increased length of gestation. A large study [202] of a Danish Birth Cohort evaluated 376 women with early preterm birth (< 34 gestational weeks, excluding preeclampsia) and 348 random controls. The average level of DHA plus EPA in plasma (measured at 9 and 25 weeks gestation) was about 27% lower in the women with early preterm birth. Levels of DHA + EPA were strongly inversely correlated with the rate of early preterm birth, plateauing at levels around 2–2.5% DHA + EPA (as % of total fatty acids). The quartile with the lowest levels of DHA + EPA had a RR = 10.3 (95% CI = 6.80–15.79, *p* < 0.0001), and the 2nd quartile had a RR = 2.86 (95% CI 1.79–4.59, *p* < 0.0001), so this was a major difference in risk of preterm birth, especially in the lowest quartile.

A meta-analysis of the effect of omega 3 fatty acid supplementation during pregnancy found several significant benefits related to preterm birth [51]. The analysis evaluated 70 RCTs involving 19,927 pregnant women comparing omega-3 LCPUFA interventions (supplements and food) compared with placebo or no omega-3. Most studies were done in upper-middle or high-income countries, and almost half of the trials included women with increased risk of adverse maternal and birth outcomes. Dosages of DHA and/or EPA varied substantially. The meta-analysis found that omega 3 supplementation significantly reduced risk of preterm birth (< 37 weeks, RR = 0.89, 95% CI = 0.81 to 0.97; 26 RCTs, 10,304 participants; high-quality evidence) and especially early preterm birth (< 34 weeks, RR = 0.58, 95% CI 0.44 to 0.77; 9 RCTs, 5204 participants; high-quality evidence). Similarly, there was a significant increase in length of gestation (mean increase of 1.67 days, 95% CI 0.95 to 2.39 days; 41 trials, 12,517 participants; moderate-quality evidence) and an increase in prolonged gestation (> 42 weeks, (RR 1.61 95% CI = 1.11 to 2.33; 5141 participants; 6 RCTs; moderate-quality evidence). There was also a reduced risk of low birth weight (RR 0.90, 95% CI 0.82 to 0.99; 15 trials, 8449 participants; high-quality evidence). Unfortunately, there was a possible small increase in large-for-gestational age infants. There was a possibly reduced risk of preeclampsia (RR = 0.84), perinatal death (RR = 0.75), and possibly fewer neonatal care admissions (RR = 0.92). In summary, this meta-analysis of many studies found that omega 3 supplementation reduced preterm birth, early preterm birth, and low birth weight, consistent with increases in length of gestation and birth weight, and possibly reduced rates of preeclampsia, perinatal death, and neonatal care admissions.

The benefits of fish oil seem to be greatest in women with low fish intake. One study [203] of 533 women found that fish oil (1300 mg EPA, 900 mg DHA) increased pregnancy duration by 7.4 days in the women with low fish intake, 4.8 days in women with medium fish intake, and little effect (minus 1.6 days) in those with high fish intake. Similar results were found in a RCT of 495 women with previous problem pregnancies [204], and in a study of 5531 women in China [69]. The latter study compared two different doses (approximately 275 mg EPA, 183 DHA vs. 1100 mg EPA, 732 DHA) and found similar benefits compared to placebo for the low-fish consumers, and no benefit for the high-fish consumers. This suggests that the lower dose (275 mg EPA, 183 DHA) was sufficient.

For women with recurring preterm birth, one study [88] of supplementation with 900 g/day of DHA and 1300 g/day of EPA resulted in a significantly lower rate of preterm birth in the supplemented group (22%) vs. the untreated group (33%), *p* = 0.05, and a significantly lower rate of early preterm birth (4.6% vs. 13.3%, *p* = 0.04). However, a similar study [205] with similar dosages found no effect.

For preeclampsia, one meta-analysis [52] analyzed 14 supplementation studies and found that omega-3 fatty acids supplementation reduced the risk of preeclampsia (RR, 0.82; 95% CI, 0.70–0.97; *p* = 0.024), and the benefit was primarily in women with low-risk pregnancies. Supplementation with DHA alone vs. DHA + EPA had similar benefits. Similarly, one study [53] found that women with preeclampsia had 17% lower levels of DHA (*p* < 0.05) in plasma at delivery compared to women with healthy pregnancies, and cord blood levels were also 17% lower.

For gestational diabetes, one meta-analysis [32] of seven RCT’s found that supplementation of omega 3 fatty acids to women with gestational diabetes substantially reduced fasting plasma glucose (standard mean difference (std. MD) = -0.56; 95% CI = -0.87 to -0.24; *p* = 0.0005), reduced homeostatic model of assessment for insulin resistance (HOMA-IR) (std. MD = -0.52; 95% CI = -0.83 to -0.21; *p* = 0.001), and high-sensitivity CRP ((std. MD = -1.14; 95% CI = -2.0 to -0.29; *p* = 0.009). There was also a possible decreased risk of macrosomia (risk ratio (RR) = 0.48; 95% CI = 0.22–1.02; *p* = 0.06) and possible reduced risk of newborns' hyperbilirubinemia (RR = 0.46; 95% CI = 0.19–1.10; *p* = 0.08). So, it appears that omega-3 fatty acid supplementation can provide significant benefits on glycemic control and inflammatory response for women with gestational diabetes, although it does not seem to help prevent it [51].

Focusing on studies in the US, Americans have been reported to have among the lowest level of DHA in human breast milk in the world (0.19%, vs. a worldwide mean of 0.32%. There were 6 RCTs on effect of DHA or DHA and EPA on preterm birth and/or gestational length. Five studies involved low doses of DHA (4 studies at doses of 300–600 mg/day of DHA and 1 study of 137 mg/day of DHA in DHA-rich eggs) and found significant improvement in rates of early preterm birth [82, 86, 90] low birth weight [82, 206] and/or gestational duration [82, 86, 89, 90, 206]. One study [207] in the US analyzed the effect of compliance with taking capsules (600 mg/day of DHA), and found that higher compliance was significantly associated with lower rates of early preterm birth, low birth weight, and very low birth weight. So, this suggests that 600 mg/day of DHA is better than lower doses for women in the US. One RCT [205] for women with recurring preterm birth involving 800 mg of DHA and 1200 mg of EPA found only slight non-significant benefits on preterm birth and gestational duration, so possibly those higher doses are less beneficial.

#### Intake

An analysis of 2003–2012 NHANES data for 788 pregnant women in the US found that they consume approximately 66 mg/day of DHA and 34 mg/day of EPA [208]. In contrast, the 2007 position paper of the American Dietetic Association and Dietitians of Canada recommend 2 servings/week of fatty fish, containing approximately 500 mg of DHA and EPA, although they do not make a recommendation on fish oil during pregnancy [209]. So, fish intake in the US is only about 20% of what is recommended.

#### Discussion

DHA supplementation is most beneficial to women with low to moderate fish intake (less than 4 servings/month) and/or a history of previous preterm birth or preeclampsia. Omega-3 supplementation does not seem to help prevent perinatal depression, but it does seem to be helpful for reducing its severity, especially with ratios of EPA/DHA above 1.5 and doses above 1 g of EPA. Review of 5 US supplementation studies suggest that 600 mg/day of DHA is better than lower dosages.

##### Quality of evidence

High.

##### Strength of recommendation to provide DHA during pregnancy

Strong.

#### Recommendation

For US women, we recommend that prenatal supplements contain approximately 600 mg of DHA, although more research is needed. This is enough to compensate for the typical decrease in DHA (as % of total fatty acids) during pregnancy, and 4 studies in the US found that dosages of 300–600 mg/day of DHA were helpful in improving rates of early preterm birth, low birth weight, and gestational duration, and one study [82] found that 600 mg/day was more effective than lower dosages. Supplementation with DHA may also reduce risk of gestational diabetes, preeclampsia, and some food allergies in infants. Women with low seafood consumption (less than 1 serving/week of fatty fish) are most likely to benefit. Women who develop prenatal or postnatal depression may benefit from adding 1000 mg or more of EPA.

#### Comparison with commercial prenatal supplements

DHA is included in 42% of prenatal supplements; when included, the median level is 200 mg (Q1: 128/Q3: 282.5) of 3 ± 1000 mg. Only 1% meet or exceed our recommendation for DHA; however, DHA or fish oil is sometimes given separately from prenatals since it is usually taken in an oil form, and a powder form would require much more volume.

### Inositol

#### Research

Inositol is a nutrient similar to glucose that is synthesized in the kidneys and present in the highest concentrations in the brain and heart. It acts as a second messenger to various hormones such as insulin, follicle stimulating hormone, and thyroid stimulating hormone. It also controls fat and sugar metabolism, nervous system cellular functions, and gene expression. Supplementation in pregnant women demonstrated improved insulin resistance in those with gestational diabetes, lower incidence of neural tube defects (NTDs) in those with folate resistant NTDs, and improve fertility in women with Polycystic Ovarian Syndrome (PCOS) (see Tables 5 and 6).

There are nine different stereoisomers of inositol, with myo-inositol being the most common natural form. Myo-inositol (MI) supplementation of 4 g per day greatly reduced gestational diabetes (GD) in at risk women [210], and reduced insulin and fasting blood glucose in women with GD [33]. A review of multiple studies of MI demonstrates its importance in regulating a variety of cellular processes, including those related to gamete development, fertilization, and early embryonic development [42].

In inositol-deficient mouse models, 70% of offspring had NTDs, causing authors to speculate that MI plays a major role in neural tube closure [113]. A case (*n* = 63) control (*n* = 102) study revealed that humans with a low maternal serum MI concentration (≤ 13.2 nmol/L) had a 2.6-fold increased risk of offspring with spina bifida, and children with spina bifida had serum MI concentrations that were 7% lower than controls [211]. One large study of pregnant women with neural tube defects (*n* = 200) vs. controls (*n* = 320), found that the maternal plasma MI concentrations in the spina bifida subgroup were 7.1% lower than controls [114]. In two small studies, women with previous NTDs (combined *n* = 31), which are at high risk of recurrent NTDs, were supplemented with MI (500 mg -1000 mg) and folic acid (5 mg) [115, 116]. In both studies, those taking the MI plus folic acid supplements had zero recurrences of NTDs.

Additionally, MI and folic acid together can restore ovarian activity and subsequent fertility in women with PCOS [34]. Women with PCOS (*n* = 98) were supplemented with MI (4 g) plus folic acid (400 mcg) or folic acid only (1.5 mg) to evaluate prevalence of gestational diabetes (GD) [212]. Prevalence of GD in the MI plus folic acid group was 17.4% vs. 54% in the folic acid only group, *p* < 0.001.

A study of 223 overweight, non-obese pregnant women found that 2000 mg/day of MI resulted in a significant decrease in gestational diabetes compared to placebo [213].

An analysis of 3 studies of myoinositol supplementation in women at risk of gestational diabetes found that 4000 mg/day of myoinositol resulted in significant reductions in the risk of preterm birth (3.4% vs. 7.6%, *p* = 0.03), macrosomia (2.1% vs 5.3%, *p* = 0.04), large for gestational age (4.8% vs. 8.9%, *p* = 0.04) and gestational diabetes (11% vs. 25%, *p* < 0.001) [214].

#### Intake

The NHANES study did not include inositol, nor is there an RDA recommendation. Daily intake is approximately 650 mg/day on a typical American diet of 1800 kcal, with ranges of about 225–1500 mg/day [215]. In addition, the kidneys produce about 2 g/day per kidney, so about 4 g total in healthy individuals, and there is also some myo-inositol production in the rest of the body [215]. A review of multiple inositol studies in humans showed zero side effects in supplementation of less than 6 g per day, with many testing 6–18 g per day. Only some mild diarrhea was reported in some subjects taking high doses up to 18 g per day. Therefore supplementation with less than 6 g appears to be safe.

#### Discussion

In determining the optimal dose for inositol, more research is needed. Doses of 4000 mg/day were enough to reduce the risk of GD in at-risk women. Doses of 500–1000 mg were enough to reduce the risk of NTD. However, in both cases it is possible that lower doses would have been sufficient.

##### Quality of evidence

High.

##### Strength of recommendation to provide vitamin A during pregnancy

Strong for women at risk of gestational diabetes PCOS, or NTD; Weak for general population.

#### Recommendation

For US women, we recommend that prenatal supplements contain approximately 500 mg of Myo-inositol, with some women at risk for gestational diabetes or with previous NTDs needing up to 4000 mg depending on diet. Note that this is a rather large volume, so although it could be taken as several capsules, it could also be consumed as a powder mixed in juice since inositol has a slightly sweet taste. This recommendation appears likely to reduce the risk of insulin resistance in gestational diabetes and NTDs in folate resistant NTDs, and improve fertility in women with PCOS.

#### Comparison with commercial prenatal supplements

Inositol is included in 17% of prenatal supplements; when included, the median level is 10 mg (Q1: 10/Q3: 20). No prenatal supplement meets nor exceeds our recommendation.

## Discussion

Vitamins are crucial dietary components needed to support human health and infant development. The levels of most vitamins decrease significantly during pregnancy, including vitamins A, C, D, K, B1, B3, B5, B6, folate, biotin, B12, resulting in increased risk of a wide range of pregnancy complications and infant health problems. Many research studies suggest that prenatal vitamin supplementation can reduce the risk of many of those problems. Note that although our review is focused on nutritional supplementation, improving diets should also be a goal to support overall health. Nutritional supplementation is not a substitute for a healthy diet but should be used to supplement a diet when needed, such as during pregnancy when the levels of many vitamins decrease substantially if not supplemented.

There is no national standard on the recommended amount of vitamins in prenatal supplements, so there is wide variation in their content.Most prenatal supplements on the market today have substantially lower levels than what we recommend. As the literature shows, supplementation below our recommendations may not adequately support a mother and her fetus. So, we believe that there is an urgent need to develop recommendations to attempt to reduce the current level of pregnancy complications and infant health problems listed in Tables 1 and 2, and which in total affect approximately half of pregnancies.

More research on this important topic is needed, but we believe that the recommendations provided here are safe and likely to significantly reduce the risk of many pregnancy complications and infant health problems. The recommendations proposed here are likely to change and improve as further research is conducted, and it is hoped that this paper will stimulate additional research to further optimize vitamin supplementation during pregnancy.

### Associations between health outcomes and vitamin status

The associations between health outcomes of mother or infant and vitamin/nutrient status are summarized in Tables 3 and 4. "Substantial Evidence" is defined as a positive meta-analysis/review or two or more statistically significant studies of the association, and a ratio of 2:1 or greater of positive to null studies. "Limited Evidence" associations are listed when there is at least one study with statistical significance, and the ratio of positive to negative studies is greater than or equal to 1:1. As can be seen in Tables 3 and 4, many maternal and infant health outcomes are associated with low levels of vitamins/nutrients.

The associations of maternal pregnancy complications with vitamin status are listed in Table 3, while the association of infant outcomes with maternal vitamin status are listed in Table 4. Both Tables 5 and 6 list the same data as Tables 1 and 2 but organized by vitamin. Altogether, there is evidence that many pregnancy and infant health problems are related to maternal vitamin/nutrient status during pregnancy, but more research is needed in some cases to verify the association.

Table 7 lists our evidenced-based recommendations for the optimal level of each vitamin/nutrient for prenatal supplements, and compares with the RDA, the NHANES average daily intake for women ages 20–39 years in the US, the tolerable upper limit, and which vitamins/nutrients decrease during pregnancy. Comparing the RDA and NHANES show that average daily intake of Vitamin D, Choline and DHA are far below the RDA. Note that the NHANES values are averages, therefore the actual values of intake for many women are less.

**Table 7:** RDA Recommendations & Daily Vitamin Intake

Nutrient	Our Daily Recommendation	RDA Recommendation for Total Daily Intake for Pregnant Women	Daily Intake (Women aged 20–39) per NHANES unless otherwise noted	Tolerable Upper Limit for Pregnant Women	Decreases significantly during pregnancy if unsupplemented
Vitamin A	1200 mcg of pre-formed vitamin A & 1000 mcg as mixed carotenoids	770 mcg	596 mcg	3000 mcg	yes
Vitamin C	200 mg	85 mg	78 mg	2000 mg	yes
Vitamin D	2000 IU	600 IU	156 IU	4000 IU	yes
Vitamin E	19 mg	15 mg	7 mg	1000 mg	unknown
Vitamin K	90 mcg	111 mcg	90 mcg	^a^	unknown
DHA	600 mg	No RDA; NIH recommends 300 mg^a^	62 mg [216]	^a^	Increases, but ratio of n-3 to n-6 decreases
Vitamin B1 (Thiamine)	6 mg	1.4 mg	1.5 mg	^a^	yes
Vitamin B2 (Riboflavin)	2 mg	1.4 mg	1.9 mg	^a^	no
Vitamin B3 (Niacin)	35 mg	18 mg	23 mg	^a^	yes
Vitamin B5 (Pantothenic Acid)	10 mg	6 mg	4 mg	^a^	yes
Vitamin B6 (Pyridoxine)	10 mg	1.9 mg	1.9 mg	100 mg	yes
Vitamin B7 (Biotin)	100 mcg	30 mcg	40 mcg^a^	^a^	yes
Vitamin B9 (Folate)	400 mcg	600 mcg	527 mcg	1000 mcg	yes
Vitamin B12	25 mcg	4.54 mcg	2.6 mcg	^a^	yes
Choline	Trimester 1 and 2: 350 mg. Trimester 3: 600 mg	450 mg	276 mg	3500 mg/day	Increased need during third trimester
Inositol	500 mg	-	2000 mg	-	unknown

^a^ Safe at high levels; Tolerable Upper Limit Not Established

The literature summarized in this review suggests that in some cases the RDA may too low, and that higher levels of intake are required to reduce the risk of pregnancy complications and infant health problems. The blood levels of most vitamins decrease substantially during pregnancy, so supplementation is needed. The recommendations in Table 7 [142, 143] appear likely to substantially reduce the many pregnancy complications and infant health problems listed in Tables 3, 4, 5 and 6.

**Fig. 1:**
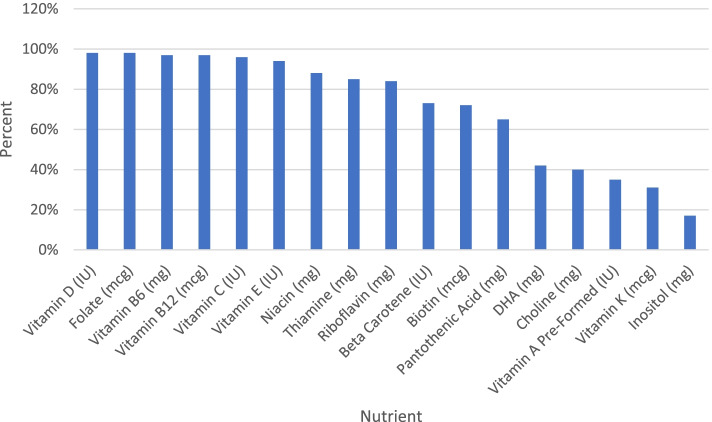
Percent of Supplements with the Nutrient

**Fig. 2:**
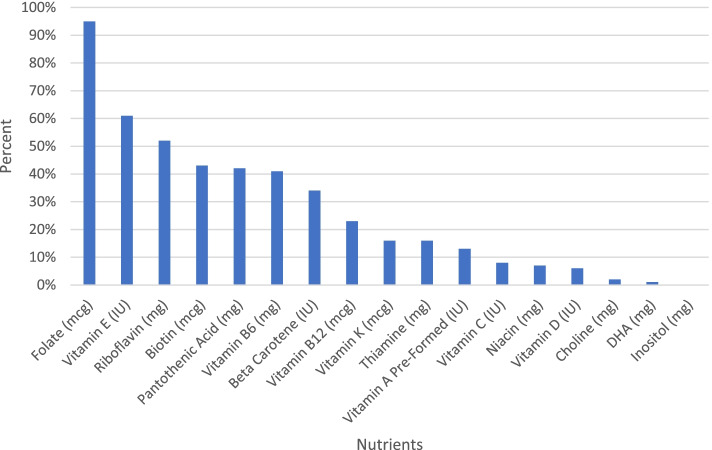
Percent of Supplements Meeting or Exceeding our Recommendation

Table 8 is a comparison of the levels of vitamins in prenatal supplements on the market compared to our evidence-based recommendations. The “average level of all supplements” is the average amount of the vitamin/nutrient, averaging across all supplements. The “average of those with the nutrient” is an average of only the supplements which contain some of that nutrient. Figure 1 shows the % of prenatal supplements that include some amount of the nutrient, even if the level is minimal. As shown in Table 8 as well as Fig. 1, almost all prenatal supplements currently on the market include folate, while most contain vitamins E, B6, B12, beta carotene, pantothenic acid, biotin and riboflavin and some contain vitamins K, A, C, thiamine, choline, DHA, and inositol. While the majority of prenatal supplements contain all or some of the nutrients recommended, many do not meet our recommendations. Almost all prenatals meet or exceed our recommendations for folate (although levels above 600 mg may increase the risk of food allergies and autism). However, as shown in Fig. 2, the levels of most other vitamins are well below our recommendations. Specifically, only 61% meet our recommendations for vitamin E, 52% meet our recommendations for riboflavin, 43% meet our recommendation for riboflavin, 42% meet our recommendations for pantothenic acid, 41% meet our recommendations for vitamin B6, 34% meet our recommendation for beta carotene, and less than 25% meet our recommendations for vitamins A (preformed), C, D, K, thiamine, niacin, B12, choline, DHA, inositol. So, most prenatals provide vitamins at levels well below our recommendations. The extensive literature discussed in this paper strongly suggests that vitamin supplementation at the levels we recommend is likely to significantly reduce the risk of many of the pregnancy and infant health conditions listed in Tables 3 and 4.

**Table 8:** Comparison of Prenatal Supplements on Market vs. our Recommendations

**Nutrients**	**Our Recommendation**	**% of Supplements with this nutrient (out of 188)**	**% Meeting or exceeding our recommendation**	**Average level of all supplements**	**Average of only those with the nutrient**	**% of the average of all supplements divided by our recommendation**	**% of the average of supplements with the nutrient divided by our recommendation**	**Range**
Vitamin A Pre-Formed (IU)	4000	35% (66)	13% (25)	1014.3 ± 1660.2	2889.14 ± 1546.9	25%	72%	0—8000
Beta Carotene (IU)	3333	73% (137)	34% (64)	2408.2 ± 2311.6	3304.7 ± 2081.0	72%	99%	0—10,000
Vitamin C (IU)	200	96% (181)	8% (15)	108.8 ± 105.1	113.0 ± 104.6	54%	57%	0—1000
Vitamin D (IU)	2000	98% (184)	6% (12)	708.6 ± 545.7	724.0 + \- 539.9	35%	36%	0 – 4200
Vitamin E (IU)	28.5	94% (176)	61% (114)	40.5 ± 47.6	43.27 ± 47.8	142%	152%	0—200
Vitamin K (mcg)	90	31% (58)	16% (31)	23.5 ± 40.0	76.2 ± 33.7	26%	85%	0—200
Thiamine (mg)	6	85% (159)	16% (30)	4.1 ± 3.9	4.8 ± 7.2	68%	81%	0—50
Riboflavin (mg)	2	84% (158)	52% (98)	4.3 ± 6.7	5.2 ± 7.0	216%	257%	0—50
Niacin (mg)	35	88% (166)	7% (13)	19.6 ± 12.1	22.1 ± 10.4	56%	63%	0 – 100
Pantothenic Acid (mg)	10	65% (123)	42% (79)	11.7 ± 23.0	17.8 ± 26.4	117%	178%	0—150
Vitamin B6 (mg)	10	97% (182)	41% (78)	10.8 ± 12.2	11.2 ± 12.1	108%	112%	0—50
Biotin (mcg)	100	72% (136)	43% (81)	165.2 ± 275.4	228.4 ± 299.8	165%	228%	0—3000
Folate (mcg)	400	98% (184)	95% (179)	854.5 ± 295.6	873.1 ± 269.4	214%	218%	0 – 2000
Vitamin B12 (mcg)	25	97% (182)	23% (43)	34.5 ± 84.8	35.6 ± 85.8	138%	142%	0—800
Choline (mg)	350	40% (75)	2% (3)	25.7 ± 71.4	64.3 ± 101.2	7%	18%	0—550
DHA (mg)	600	42% (79)	1% (2)	94.2 ± 150.4	224.1 ± 156.4	16%	37%	0 – 1000
Inositol (mg)	500	17% (32)	0% (0)	4.5 ± 19.7	26.5 ± 41.2	1%	5%	0—150

## Limitation Section

1. This paper provides a general summary of important research on the optimal level of supplementation for each vitamin. However, a systematic review of each vitamin should be conducted to confirm the recommendations made here.
2. There is a wide variation in nutritional intake among women in the US, and the recommendations here are for the general population. Women with especially poor diets or pre-existing health conditions may need additional or different supplementation. Women with very good diets and in good health may need less supplementation.
3. The current recommendations assume that no individualized testing of nutritional status is conducted. Individual assessments of dietary intake and biochemical measures of nutritional status could be used to tailor recommendations for an individual.
4. Prenatal supplements are intended to supplement, and not replace, a healthy diet. Healthy diets should be encouraged especially during pregnancy.
5. Most supplementation studies were started after conception, but some women start supplements prior to conception, and so studies prior to conception are needed and may show different benefits.
6. Most supplementation studies involved only a single vitamin. There is a strong need for additional studies in which the effects of a multi-vitamin supplement are assessed in a large-scale clinical trial, including assessments of dietary intake, biochemical measures of nutritional status, assessments of pregnancy/birth complications, and short and long-term assessments of children’s health. We are in the process of planning such a study.

## Conclusions and implications

The levels of most vitamins decrease significantly during pregnancy, and low levels of vitamins are associated with a wide range of pregnancy and infant health complications. Therefore, vitamin supplementation is important during pregnancy to prevent a wide range of problems. Unfortunately, there is no national standard for the content of prenatal supplements, so they vary widely in their content, and many contain only a few vitamins. This paper provides evidence-based recommendations for the optimal level of vitamins in prenatal supplements to reduce the risk of a wide range of pregnancy and infant health problems.

It is hoped that the current recommendations will help women and their physicians/nutritionists to choose the best prenatal supplements. Further, it is hoped that these recommendations will encourage manufacturers to produce higher-quality supplements. Finally, the present recommendations will hopefully stimulate further research and discussion, leading to even better recommendations in future.

The present recommendations were used to rate over 180 prenatal supplements, and those ratings are available in a smartphone app, Prenatal Rater, which is available for free from the app stores for Android and iOS systems.

## Supplementary Information

**Additional file 1:Supplemental Table 1 (S1).** Additional information about research studies discussed.

## Data Availability

Not applicable.
