# Supplementary material for: Evidence based recommendations for an optimal prenatal supplement for women in the US: vitamins and related nutrients
Source: Matern Health Neonatol Perinatol. 2022 Jul 11;8:4. doi: 10.1186/s40748-022-00139-9 (PMC9275129; doi:10.1186/s40748-022-00139-9)
Supplement: Supplementary file 1 — Additional file 1:Supplemental Table 1 (S1). Additional information about research studies discussed. [file 40748_2022_139_MOESM1_ESM.docx]

**Supplemental Table 1 (S1): Additional information about research studies discussed**

| **Vitamins(s) Reviewed** | **Reference** | **Country** | **Number of Participants** | **Study Type** | **Link** |
| --- | --- | --- | --- | --- | --- |
| Vitamin A,  Vitamin D, B2 (Riboflavin), B6 (Pyridoxine), B9 (Folate), B12 (Cobalamin) | Bruinse 1995 | Netherlands | 116 | O | https://www.sciencedirect.com  /science/article/pii/002822439502150Q |
| Vitamin A, Vitamin E, B1 (Thiamine), B2 (Riboflavin), B6 (Pyroxidine), B9 (Folate), B12 (Cobalamin) | Cikot 2001 | Netherlands | 102 | O | https://pubmed.ncbi.nlm.nih.gov/11227033/ |
| Vitamin A, B1 (Thiamine), B2 (Riboflavin), B3 (Niacin), B7 (Biotin) | Baker 1975 | United States | 174 | O | <https://academic.oup.com/aj>  cn/article-abstract/28/1/59/4716328 |
| Vitamin A, Vitamin C, Vitamin E, B1 (Thiamine), B2 (Riboflavin), B3 (Niacin), B5 (Pantothenic Acid), B6 (Pyridxine), B7 (Biotin), B12 (Cobalamin) | Baker 2002 | New Jersy, United States | 563 | O | [https://pubmed.ncbi.nlm.nih.gov/11838885/](about:blank) |
| Vitamin A | WHO | Worldwide | N/A | R | <http://apps.who.int/iris/bitstream/handle/>  10665/44110/9789241598019_  eng.pdf;jsessionid=D63920AD0  EF940919B459D6EAF49A255?sequence=1 |
| Vitamin A, Vitamin E | Ziari 1996 | Maiduguri and Bauchi, Nigeria | 72 | O | [https://pubmed.ncbi.nlm.nih.gov/8863947/](about:blank) |
| Vitamin A | Thorne-Lyman 2012 | Worldwide | 23 Studies | M/R |  |
| Vitamin A | Reifen 2001 | N/A | N/A | R |  |
| Vitamin A | Underwood 1994 | N/A | N/A | R |  |
| Vitamin A | Checkley 2010 | Nepali | 44646 | T | <https://www.nejm.org/doi/10>.  1056/NEJMoa0907441?url_ver=Z39.88-2003&rfr_id=ori%3Arid%3Acrossref.  org&rfr_dat=cr_pub++0www.ncbi.nlm.nih.gov |
| Vitamin A, Vitamin C, Vitamin E, B6 (Pyridoxine) | Krapels 2004 | Nikmegen, Netherlands | 355 | O | <https://academic.oup.com>  /jn/article/134/11/3106/4688446 |
| Vitamin A, Vitamin E, B2 (Riboflavin), B3 (Niacin), B12 (Cobalamin), Choline | Shaw 2010 | California, United States | 1001 | O | <https://www.ncbi.nlm.nih>.  gov/pmc/articles/PMC2849796/ |
| Vitamin A | Kizer 1990 | N/A | N/A | R |  |
| Vitamin C | Casanueva 2005 | Mexico | 10 | T | [https://www.medigraphic.com/cgi-bin/new/resumenI.cgi?IDARTICULO=4955](about:blank) |
| Vitamin C, Vitamin E | Rumbold 2009 | Australia | 24300 (29 different studies) | R | [https://pubmed.ncbi.nlm.nih.gov/26415762/](about:blank) |
| Vitamin C | Ghomian 2013 | Iran | 170 | T | <https://www.ncbi.nlm.nih>.  gov/pmc/articles/PMC3652497/ |
| Vitamin C | Zamini 2013 | Iran | 60 | T | <https://www.ncbi.nlm.nih.gov/pmc/>  articles/PMC3808946/ |
| Vitamin C | Kiondo 2014 | Uganda | 932 | T | [https://pubmed.ncbi.nlm.nih.gov/25142305/](about:blank) |
| Vitamin C | Hans 2010 | Italy | 384 | T | [https://pubmed.ncbi.nlm.nih.gov/21293742/](about:blank) |
| Vitamin C | Ochoa-Brust 2007 | Mexico | 110 | T | https://pubmed.ncbi.nlm.nih.gov/17611821/ |
| Vitamin C | Rumbold 2008 | Australia | 6533 (10 different studies) | R | [https://pubmed.ncbi.nlm.nih.gov/18254042/](about:blank) |
| Vitamin C | Chappell 2002 | United Kingdom | 182 | T | [https://pubmed.ncbi.nlm.nih.gov/12237663/](about:blank) |
| Vitamin C, Vitamin E | Xu 2010 | Canada | 2647 | T | [https://pubmed.ncbi.nlm.nih.gov/20207239/](about:blank) |
| Vitamin C | McEvoy 2014 | United States | 179 | T | [https://pubmed.ncbi.nlm.nih.gov/24838476/](about:blank) |
| B5 (Pantothenic Acid) | Haggarty 2009 | United Kingdom | 1461 | O | [https://pubmed.ncbi.nlm.nih.gov/19682400/](about:blank) |
| Vitamin D | Johnson 2010 | United States | 494 | O | [https://pubmed.ncbi.nlm.nih.gov/20640974/](about:blank) |
| Vitamin D | Andersen 2015 | Denmark | 1683 | O | [https://pubmed.ncbi.nlm.nih.gov/26178723/](about:blank) |
| Vitamin D | Wagner 2016 | United States | 509 | O | [https://pubmed.ncbi.nlm.nih.gov/26554936/](about:blank) |
| Vitamin D | Bodnar 2007 | United States | 274 | O | <https://www.ncbi.nlm.nih>.  gov/pmc/articles/PMC4288954/ |
| Vitamin D | Baca 2016 | United States | 2327 | O | [https://pubmed.ncbi.nlm.nih.gov/27818017/](about:blank) |
| Vitamin D | Merewood 2009 | United States | 253 | O | [https://pubmed.ncbi.nlm.nih.gov/19106272/](about:blank) |
| Vitamin D | Haugen 2009 | Norway | 22423 | O | [https://pubmed.ncbi.nlm.nih.gov/19451820/](about:blank) |
| Vitamin D | Yu 2009 | United Kingdom | 180 | T | [https://pubmed.ncbi.nlm.nih.gov/18771564/](about:blank) |
| Vitamin D | Wagner 2013 | United States | 504 | T | [https://pubmed.ncbi.nlm.nih.gov/23314242/](about:blank) |
| Vitamin D | Whitehouse 2012 | Australia | 743 | O | [https://pubmed.ncbi.nlm.nih.gov/22331333/](about:blank) |
| Vitamin D | Vinkhuyzen 2018 | Netherlands | 9778 | O | [https://pubmed.ncbi.nlm.nih.gov/27895322/](about:blank) |
| Vitamin D | Fernell 2015 | Sweden | 58 | O | <https://molecularautism.biomed>  central.com/articles/10.1186/2040-2392-6-3 |
| Vitamin D | Morales 2012 | Spain | 1820 | O | <https://pediatrics.aappublications>.  org/content/130/4/e913.1.short |
| Vitamin D, Vitamin E | Beckhaus 2015 | worldwide | 32 Studies | R/M | [https://pubmed.ncbi.nlm.nih.gov/26296633/](about:blank) |
| Vitamin D | Zoksy 2014 | Auatralia | 2834 | O | [https://pubmed.ncbi.nlm.nih.gov/24601713/](about:blank) |
| Vitamin D | Wolsk 2017 | United States | 712 | T | [https://pubmed.ncbi.nlm.nih.gov/28285844/](about:blank) |
| Vitamin D | Baker 2010 | United States | 3992 | O | <https://www.ncbi.nlm.nih.gov/>  pmc/articles/PMC2968727/ |
| Vitamin D | Litonjua 2016 | United States | 876 | T | [https://pubmed.ncbi.nlm.nih.gov/26813209/](about:blank) |
| Vitamin E | Ley 2013 | Canada | 205 | O | [https://pubmed.ncbi.nlm.nih.gov/24065066/](about:blank) |
| Vitamin E | Bártfai 2012 | Hungary | 38151 | T | <https://www.tandfonline.com/doi/abs/>  10.3109/14767058.2011.  587060?journalCode=ijmf20 |
| Vitamin E | Rumbold 2015 | Worldwide | (17 Studies) | M | [https://pubmed.ncbi.nlm.nih.gov/26343254/](about:blank) |
| Vitamin E | Spinnato 2007 | Brazil | 739 | T | [https://pubmed.ncbi.nlm.nih.gov/18055726/](about:blank) |
| Vitamin E | Rubmbold 2006 | Australia | 1877 | T | [https://pubmed.ncbi.nlm.nih.gov/16641396/](about:blank) |
| Vitamin E, B6 (Pyridoxine) | Ronnenberg 2002 | United States | 423 | O | [https://pubmed.ncbi.nlm.nih.gov/12450907/](about:blank) |
| Vitamin K | Greer 2001 | United States | 119 | O | [https://pubmed.ncbi.nlm.nih.gov/11787707/](about:blank) |
| Vitamin K | American Academy of Pediatrics Committee on Fetus and Newborn | United States | N/A | R | [https://pubmed.ncbi.nlm.nih.gov/12837888/](about:blank) |
| Vitamin K | Dituri 2012 | Rome | 53 | T | [https://pubmed.ncbi.nlm.nih.gov/22280352/](about:blank) |
| Vitamin K | Witt 2016 | Europe | 290 | T | [https://pubmed.ncbi.nlm.nih.gov/27244818/](about:blank) |
| Vitamin K | Greer 1997 | United States | 20: stage 1; 22: stage 2 | T | [https://pubmed.ncbi.nlm.nih.gov/8989344/](about:blank) |
| Vitamin K | Jans 2014 | Belgium | 49 | T | [https://pubmed.ncbi.nlm.nih.gov/25264330/](about:blank) |
| Vitamin K | Kazzi 1990 | United States | 78 | T | [https://pubmed.ncbi.nlm.nih.gov/2304704/](about:blank) |
| Vitamin K | Crowther 2001 | Australia | 5 trials, > 420 women | T | [https://pubmed.ncbi.nlm.nih.gov/11279686/](about:blank) |
| Vitamin K | Israels 1997 | Canada | 18 | T | [https://pubmed.ncbi.nlm.nih.gov/9190038/](about:blank) |
| Vitamin K | Saxena 2001 | N/A | N/A | R | [https://pubmed.ncbi.nlm.nih.gov/11321042/](about:blank) |
| Vitamin K | Roman 2002 | Great Britain; Germany | 2431; 6338 control | M | [https://pubmed.ncbi.nlm.nih.gov/11857013/](about:blank) |
| B1 (Thiamine) | Bakker 2000 | Netherlands | N/A | R | [https://pubmed.ncbi.nlm.nih.gov/11021334/](about:blank) |
| B1 (Thiamine) | Heinze 1990 | West Germany | 72 | O | [https://pubmed.ncbi.nlm.nih.gov/2333720/](about:blank) |
| B1 (Thiamine) | Chandler 2012 | United States | 954 cases, 6268 controls | O | [https://pubmed.ncbi.nlm.nih.gov/22933447/](about:blank) |
| B1 (Thiamine) | Bell 1979 | N/A | N/A | A | [https://pubmed.ncbi.nlm.nih.gov/479952/](about:blank) |
| B2 (Riboflavin) | Powers 1985 | Africa | 81 | T | [https://pubmed.ncbi.nlm.nih.gov/4019262/](about:blank) |
| B2 (Riboflavin) | Bates 1981 | Africa & England | 215 | T | [https://pubmed.ncbi.nlm.nih.gov/7234718/](about:blank) |
| B2 (Riboflavin) | Elsen 2012 | Africa & South America | 414 | T | [https://pubmed.ncbi.nlm.nih.gov/25308984/](about:blank) |
| B2 (Riboflavin) | Ma 2008 | China | 366 | T | [https://pubmed.ncbi.nlm.nih.gov/18806105/](about:blank) |
| B2 (Riboflavin) | Suprapto 2002 | Indoneisa | 103 | T | [https://pubmed.ncbi.nlm.nih.gov/12495257/](about:blank) |
| B2 (Riboflavin) | Graham 2007 | Nepal | 6645 | T | [https://pubmed.ncbi.nlm.nih.gov/17490976/](about:blank) |
| B3 (Niacin) | Groenen 2004 | Netherlands | 128 | O | [https://pubmed.ncbi.nlm.nih.gov/15295338/](about:blank) |
| B5 (Pantothenic Acid) | Ishiguro 1962 | Japan | 13 | T |  |
| B5 (Pantothenic Acid) | Cohenour 1972 | United States | 30 | O |  |
| B5 (Pantothenic Acid) | Song 1985 | United States | 26 | O | [https://pubmed.ncbi.nlm.nih.gov/3968356/](about:blank) |
| B5 (Pantothenic Acid) | Lagiou 2005 | United States | 222 | O | [https://pubmed.ncbi.nlm.nih.gov/15309438/](about:blank) |
| B5 (Pantothenic Acid) | Watson 2010 | New Zealand | 504 | O | [https://pubmed.ncbi.nlm.nih.gov/19920847/](about:blank) |
| B5 (Pantothenic Acid) | Baker 1977 |  | 100 | O | [https://pubmed.ncbi.nlm.nih.gov/910841/](about:blank) |
| B6 (Pyridoxine) | Schuster 1984 | United States | 46 | T | [https://pubmed.ncbi.nlm.nih.gov/6726466/](about:blank) |
| B6 (Pyridoxine) | Chittumma 2007 | Thailand | 126 | T | [https://pubmed.ncbi.nlm.nih.gov/17621727/](about:blank) |
| B6 (Pyridoxine) | Ensiyeh 2009 | Iran | 70 | T | [https://pubmed.ncbi.nlm.nih.gov/18272271/](about:blank) |
| B6 (Pyridoxine) | Wibowo 2012 | Indonesia | 60 | T | [https://pubmed.ncbi.nlm.nih.gov/22189065/](about:blank) |
| B6 (Pyridoxine) | McCullough 1990 | Egypt | 70 | O | [https://pubmed.ncbi.nlm.nih.gov/2349921/](about:blank) |
| B6 (Pyridoxine) | Sahakian 1991 | United States | 59 | T | [https://pubmed.ncbi.nlm.nih.gov/2047064/](about:blank) |
| B6 (Pyridoxine) | Mathews 2014 | Ireland | 5049; 37 trials | T | [https://pubmed.ncbi.nlm.nih.gov/24659261/](about:blank) |
| B6 (Pyridoxine) | Czeizel 2004 | Hungary | 22843 + 38151 control | T | [https://pubmed.ncbi.nlm.nih.gov/15357625/](about:blank) |
| B6 (Pyridoxine) | Chang 1999 | Taiwan | 209 | O | [https://pubmed.ncbi.nlm.nih.gov/10575635/](about:blank) |
| B6 (Pyridoxine) | Salam 2015 | Pakistan | 1646 | T | [https://pubmed.ncbi.nlm.nih.gov/26039815/](about:blank) |
| B6 (Pyridoxine) | Hillman 1962 | United States | 540 | T | https://academic.oup.com/ajcn/article-  abstract/10/6/512/4829555?  redirectedFrom=fulltext |
| B7 (Biotin) | Mock 2002 | United States | 26 | T | [https://pubmed.ncbi.nlm.nih.gov/11815321/](about:blank) |
| B7 (Biotin) | Mock 2009 | United States | 22 | O | [https://pubmed.ncbi.nlm.nih.gov/19056637/](about:blank) |
| B7 (Biotin) | Perry 2014 | United States | 75 | T | [https://pubmed.ncbi.nlm.nih.gov/25122647/](about:blank) |
| B7 (Biotin) | Watanabe 1983 | Japan | N/A | A | [https://pubmed.ncbi.nlm.nih.gov/2274895/](about:blank) |
| B7 (Biotin) | Watanabe 1983 | Japan | N/A | A | [https://pubmed.ncbi.nlm.nih.gov/6827377/](about:blank) |
| B7 (Biotin) | Takechi 2008 | Japan | N/A | C | [https://pubmed.ncbi.nlm.nih.gov/18356320/](about:blank) |
| B7 (Biotin) | Mock 2014 |  | N/A | R | <https://www.ncbi.nlm.nih>.  gov/pmc/articles/PMC4230206/ |
| B9 (Folate) | Willoughby 1966 | Scotland | 350 | T | <https://www.ncbi.nlm.nih>.  gov/pmc/articles/PMC1944952/ |
| B9 (Folate) | Lassi 2013 | Pakistan | 17771; 31 trials | R | [https://pubmed.ncbi.nlm.nih.gov/23543547/](about:blank) |
| B9 (Folate) | Daly 1995 | Ireland | N/A | R | [https://pubmed.ncbi.nlm.nih.gov/7474275/](about:blank) |
| B9 (Folate) |  | India | 279 | T |  |
| B9 (Folate) | Berry 1999 | China | 285536 | T | [https://pubmed.ncbi.nlm.nih.gov/10559448/](about:blank) |
| B9 (Folate) | Laurence 1981 | United Kingdom | 60 | T | [https://pubmed.ncbi.nlm.nih.gov/6786536/](about:blank) |
| B9 (Folate) |  | 7 countries | 1837 | T |  |
| B9 (Folate) | Toriello 2005 | N/A | N/A | R |  |
| B9 (Folate) | Vergel 1990 | Cuba | 81 | T | [https://pubmed.ncbi.nlm.nih.gov/2343028/](about:blank) |
| B9 (Folate) | Werler 1993 | United States/Canada | 3051 | T | [https://pubmed.ncbi.nlm.nih.gov/8437302/](about:blank) |
| B9 (Folate) | Wilson 2015 |  | 1195 | T | [https://pubmed.ncbi.nlm.nih.gov/26334606/](about:blank) |
| B9 (Folate) | Pfeiffer 2012 | United States | 23359 | O | <https://www.ncbi.nlm.nih>.  gov/pmc/articles/PMC3327747/ |
| B9 (Folate) | Ingrid 2006 | worldwide | 41 studies | M | [https://pubmed.ncbi.nlm.nih.gov/17022907/](about:blank) |
| B9 (Folate) | Li 2019 | China | 10 studies | M | [https://pubmed.ncbi.nlm.nih.gov/31171831/](about:blank) |
| B9 (Folate) | Hodgetts 2015 | United Kingdom | 111736 = population study; 188796 systematic review | R | [https://pubmed.ncbi.nlm.nih.gov/25424556/](about:blank) |
| B9 (Folate) | Roth 2011 | Norway | 38954 | T | [https://pubmed.ncbi.nlm.nih.gov/21990300/](about:blank) |
| B9 (Folate) | Li 2019 | United States | 37 reviewed; 6 analyzed | R/M | [https://pubmed.ncbi.nlm.nih.gov/31319515/](about:blank) |
| B9 (Folate), B12 (Cobalamin) | Raghavan 2018 | United States | 1257 | T | [https://pubmed.ncbi.nlm.nih.gov/28984369/](about:blank) |
| B9 (Folate) | Raghavan 2020 | United States | 567 | O | [https://pubmed.ncbi.nlm.nih.gov/32844208/](about:blank) |
| B9 (Folate) | McGowan 2019 | United States | 1394 | O | [https://pubmed.ncbi.nlm.nih.gov/31252026/](about:blank) |
| B12 (Cobalamin) | Koebnick 2002 | Germany | 39 | O | [https://pubmed.ncbi.nlm.nih.gov/12029010/](about:blank) |
| B12 (Cobalamin) | Yajnik 2008 | India | 700 | O | [https://pubmed.ncbi.nlm.nih.gov/17851649/](about:blank) |
| B12 (Cobalamin) | Green 2016 | India | 19 studies | R | [https://pubmed.ncbi.nlm.nih.gov/27146890/](about:blank) |
| B12 (Cobalamin) | Fothergill | United States | 1173 | M | <https://faseb.onlinelibrary.wiley>.  com/doi/abs/10.1096/fasebj.31.1  _supplement.lb439 |
| B12 (Cobalamin) | Ray 2008 | Canada | 10622 | O | [https://pubmed.ncbi.nlm.nih.gov/18337285/](about:blank) |
| B12 (Cobalamin) | Ray 2007 | Canada | 89 | O | [https://pubmed.ncbi.nlm.nih.gov/17474166/](about:blank) |
| B12 (Cobalamin) | Molloy 2009 | Ireland | 1179 | O | [https://pubmed.ncbi.nlm.nih.gov/19255021/](about:blank) |
| B12 (Cobalamin) | Mills 1995 | United States | 404 | O | [https://pubmed.ncbi.nlm.nih.gov/7741859/](about:blank) |
| B12 (Cobalamin) | Wald 1996 | United Kingdom, Hungary, Israel, Australia, Canada and Russia | 135 | R | [https://pubmed.ncbi.nlm.nih.gov/8605127/](about:blank) |
| B12 (Cobalamin) | Jackson 1967 | Scotland | 8 | T | [https://pubmed.ncbi.nlm.nih.gov/4168374/](about:blank) |
| B12 (Cobalamin) | Hall 1968 | Scotland | 5 | T | [https://pubmed.ncbi.nlm.nih.gov/5697363/](about:blank) |
| B12 (Cobalamin) | Bennett 2001 | Israel | 14 | O | [https://pubmed.ncbi.nlm.nih.gov/11304860/](about:blank) |
| B12 (Cobalamin) | Reznikoff-Etiévant 2002 | France | 10 | O | [https://pubmed.ncbi.nlm.nih.gov/12206930/](about:blank) |
| B12 (Cobalamin) | Hübner 2008 | Syria | 75 (32 controls) | O | [https://pubmed.ncbi.nlm.nih.gov/18636794/](about:blank) |
| B12 (Cobalamin) | Rogne 2017 | worldwide | 22 studies; 18 for M | R/M | [https://pubmed.ncbi.nlm.nih.gov/28108470/](about:blank) |
| B12 (Cobalamin) | Finkelstein 2015 | United States | 1 trial | R | [https://pubmed.ncbi.nlm.nih.gov/26374177/](about:blank) |
| B12 (Cobalamin) | Mardali 2021 | Europe, Asia, America, Africa | 19 articles; 3211 participants | R/M | [https://pubmed.ncbi.nlm.nih.gov/33001182/](about:blank) |
| B12 (Cobalamin) | Bae 2015 | United States | 54 | T | [https://pubmed.ncbi.nlm.nih.gov/25995278/](about:blank) |
| B12 (Cobalamin) | Duggan 2014 | India | 366 | T | [https://pubmed.ncbi.nlm.nih.gov/24598885/](about:blank) |
| B12 (Cobalamin) | Hollowood-Jones 2020 | United States | 59 | T | [https://pubmed.ncbi.nlm.nih.gov/33317469/](about:blank) |
| Choline | Caudill 2010 | N/A | N/A | R | [https://pubmed.ncbi.nlm.nih.gov/20656095/](about:blank) |
| Choline | Yan 2013 | United States | 47 | T | [https://pubmed.ncbi.nlm.nih.gov/24132975/](about:blank) |
| Choline | Jiang 2014 | United States |  | R | [https://pubmed.ncbi.nlm.nih.gov/24680198/](about:blank) |
| Choline | Boeke 2013 | United States | 1896 | T | [https://pubmed.ncbi.nlm.nih.gov/23425631/](about:blank) |
| Choline | Yan 2012 | United States | 47 | T | [https://pubmed.ncbi.nlm.nih.gov/22418088/](about:blank) |
| Choline | Strupp 2012 | United States | N/A | R | [https://pubmed.ncbi.nlm.nih.gov/26391046/](about:blank) |
| Choline | Ross 2013 | United States | 100 | T | [https://pubmed.ncbi.nlm.nih.gov/23318559/](about:blank) |
| DHA | Burdge 2002 | United States | 6 | T | [https://pubmed.ncbi.nlm.nih.gov/12323090/](about:blank) |
|  | Wilson 2019 | Australia | 13 studies | M | [https://pubmed.ncbi.nlm.nih.gov/31088623/](about:blank) |
| DHA | Vlaardingerbroek 2004 | Netherlands | 184 | O | [https://pubmed.ncbi.nlm.nih.gov/15519495/](about:blank) |
| DHA | Al 1995 | Netherlands | 110 | O | [https://pubmed.ncbi.nlm.nih.gov/7547829/](about:blank) |
| DHA | Harris 2015 | United States | 564 | T | [https://pubmed.ncbi.nlm.nih.gov/26413500/](about:blank) |
| DHA | Lin 2017 | Taiwan | 12 studies | R/M | [https://pubmed.ncbi.nlm.nih.gov/28410627/](about:blank) |
| DHA | Hibbeln 2002 | United States; 23 countries | 14532 subjects, 41 studies | O | [https://pubmed.ncbi.nlm.nih.gov/12103448/](about:blank) |
| DHA | Zhang 2020 | worldwide | 638 | M | <https://www.ncbi.nlm.nih>.  gov/pmc/articles/PMC7299975/ |
| DHA | Liao 2019 | China | 26 studies; 2160 participants | R/M | [https://pubmed.ncbi.nlm.nih.gov/31383846/](about:blank) |
| DHA | Middleton 2018 | Australia | 19927 | M | [https://pubmed.ncbi.nlm.nih.gov/30480773/](about:blank) |
| DHA | Ciesielski 2019 | 184 Countries | N/A | M | [https://bmjopen.bmj.com/content/9/4/e027249](about:blank) |
| DHA | Olsen 1989 | Denmark | 29 | O | [https://pubmed.ncbi.nlm.nih.gov/2706041/](about:blank) |
| DHA | Olsen 1991 | Denmark | 99 | O | [https://pubmed.ncbi.nlm.nih.gov/1827949/](about:blank) |
| DHA | Klebanoff 2011 | United States | 852 | T | [https://pubmed.ncbi.nlm.nih.gov/21508745/](about:blank) |
| DHA | Olsen 2018 | Denmark | 724 | O | [https://pubmed.ncbi.nlm.nih.gov/30082226/](about:blank) |
| DHA | Olsen 2007 | Denmark | 533 | T | [https://pubmed.ncbi.nlm.nih.gov/1349049/](about:blank) |
| DHA | Olsen 2019 | Europe | 968 | T | [https://pubmed.ncbi.nlm.nih.gov/17299499/](about:blank) |
| DHA | Olsen 2000 | China | 5531 | T | [https://pubmed.ncbi.nlm.nih.gov/31387119/](about:blank) |
| DHA | Olsen 2000 | Europe | 1477 | T | [https://pubmed.ncbi.nlm.nih.gov/10740336/](about:blank) |
| DHA | Harper 2010 | United States | 852 | T | [https://pubmed.ncbi.nlm.nih.gov/20093894/](about:blank) |
| DHA | Bakouei 2020 | Iran | 14 studies | R/M | [https://pubmed.ncbi.nlm.nih.gov/32039806/](about:blank) |
| DHA | Kulkarni 2010 | India | 117 | O | [https://pubmed.ncbi.nlm.nih.gov/20224572/](about:blank) |
| DHA | Gao 2020 | China | 7 articles | R/M | [https://pubmed.ncbi.nlm.nih.gov/30231792/](about:blank) |
| DHA | Juber 2017 | United States | 84 | O | [https://pubmed.ncbi.nlm.nih.gov/28149321/](about:blank) |
| DHA | Carlson 2013 | United States | 350 | T | [https://pubmed.ncbi.nlm.nih.gov/23426033/](about:blank) |
| DHA | Miller 2006 | United States | 115 | T |  |
| DHA | Keenan 2014 | United States | 64 | T | [https://pubmed.ncbi.nlm.nih.gov/25415158/](about:blank) |
| DHA | Smuts 2003 | United States | 291 | T | [https://pubmed.ncbi.nlm.nih.gov/12636950/](about:blank) |
| DHA | Carlson 2013 | United States | 345 | T | [https://pubmed.ncbi.nlm.nih.gov/30392575/](about:blank) |
| DHA | Nordgren 2017 | United States | 6478 | O | [https://pubmed.ncbi.nlm.nih.gov/28245632/](about:blank) |
| DHA | Zhang 2018 | United States | 11465 | O |  |
| Inositol | Kris-Etherton 2007 | N/A | N/A | R | [https://pubmed.ncbi.nlm.nih.gov/23327487/](about:blank) |
| Inositol | Matarrelli 2013 | Italy | 74 | T | [https://pubmed.ncbi.nlm.nih.gov/21414183/](about:blank) |
| Inositol | Corrado 2011 | Italy | 69 | T | [https://pubmed.ncbi.nlm.nih.gov/21835536/](about:blank) |
| Inositol | Carlomagno 2011 | N/A | N/A | R | [https://pubmed.ncbi.nlm.nih.gov/27324558/](about:blank) |
| Inositol | Greene 2017 | United Kingdom |  | R | [https://pubmed.ncbi.nlm.nih.gov/15173422/](about:blank) |
| Inositol | Groenen 2004 | Netherlands | 287 | O | [https://pubmed.ncbi.nlm.nih.gov/24465924/](about:blank) |
| Inositol | Guan 2014 | China | 520 | O | [https://pubmed.ncbi.nlm.nih.gov/26847388/](about:blank) |
| Inositol | Greene 2016 | United Kingdom | 99 | T | [https://pubmed.ncbi.nlm.nih.gov/21956977/](about:blank) |
| Inositol | Cavalli 2011 | Italy | 12 | T | [https://pubmed.ncbi.nlm.nih.gov/17952759/](about:blank) |
| Inositol | Papaleo 2011 | Italy | 25 | T | [https://pubmed.ncbi.nlm.nih.gov/22122627/](about:blank) |
| Inositol | D'Anna 2012 | Italy | 98 | O | [https://pubmed.ncbi.nlm.nih.gov/33238798/](about:blank) |
| Inositol | Vitale 2020 | Italy | 223 | T | [https://pubmed.ncbi.nlm.nih.gov/29859136/](about:blank) |
| Inositol | Santamaria 2018 | Italy | 595 | M | [https://pubmed.ncbi.nlm.nih.gov/23764390/](about:blank) |
| Inositol | Croze 2013 | N/A | N/A | R | [https://pubmed.ncbi.nlm.nih.gov/29597261/](about:blank) |

Supplemental Table 1 gives additional information about the studies included. M represents a meta-analysis, R represents reviews, O represents observational study, T represents a treatment study, A represents an animal study, and C represents a cell culture study.
